# Immunometabolic reprogramming of central and peripheral immune cells in mental disorders: mechanisms and therapeutic implications

**DOI:** 10.3389/fimmu.2026.1712425

**Published:** 2026-08-04

**Authors:** Kexin Lin, Peipei Wang, Shen Li, Chunling Ma, Di Wen, Xia Wang, Linchuan Liao

**Affiliations:** 1 Department of Forensic Toxicological Analysis, West China School of Basic Medical Sciences and Forensic Medicine, Sichuan University, Chengdu, China; 2 Department of Immunology, West China School of Basic Medical Sciences and Forensic Medicine, Sichuan University, Chengdu, China; 3 Department of Biotherapy, West China Hospital and State Key Laboratory of Biotherapy, Sichuan University, Chengdu, China; 4 College of Forensic Medicine, Hebei Medical University, Hebei Key Laboratory of Forensic Medicine, Collaborative Innovation Center of Forensic Medical Molecular Identification, Shijiazhuang, China

**Keywords:** cellular metabolism, immune cells, major depressive disorder, neurodegenerative diseases, schizophrenia

## Abstract

Immune cells are those involved in or related to immune responses, found throughout immune organs and the body. The discipline of immunometabolism, which merges immunology with metabolism, has gained significant attention in recent years. This emerging field focuses on the metabolic processes and mechanisms of various immune cells, aiming to uncover how these cells’ metabolism influences disease onset and progression. Research in immunometabolism spans several diseases, including chronic inflammatory conditions, infectious diseases, cardiovascular disorders, and cancer, highlighting the critical role of immune cell metabolism in these diseases. Mental illnesses, characterized by brain function abnormalities due to biological, psychological, and environmental factors, lead to impairments in cognitive, emotional, volitional, and behavioral functions. Conditions such as schizophrenia, neurodegenerative diseases (Alzheimer’s disease AD, Parkinson’s disease PD), anxiety, and depression are associated with significant metabolic changes. The intersection of neuroimmunology and immunometabolism has become a focal point for understanding the regulation of mental illnesses by immune cells’ metabolic alterations. This review systematically examines how metabolic reprogramming of central and peripheral immune cells contributes to the pathogenesis of mental disorders, and critically evaluates emerging therapeutic strategies targeting these immunometabolic pathways—including pharmacological modulators (HK2 inhibitors, kynurenine pathway modulators, CD38 checkpoint targeting), lifestyle interventions (ketogenic diet, exercise), and their translational challenges. By integrating mechanistic insights with therapeutic perspectives, this review aims to provide fresh insights into disease mechanisms and inform the development of precision diagnostic and therapeutic approaches for mental disorders.

## 1 Introduction

Mental disorders, including schizophrenia, major depressive disorder (MDD), bipolar disorder, and neurodegenerative diseases [e.g., Alzheimer’s disease (AD), Parkinson’s disease (PD)], are among the leading causes of disability and mortality worldwide (1, 2). The etiologies of these disorders are extremely complex, resulting from long-term interactions among genetic susceptibility, neurodevelopmental abnormalities, environmental factors (such as infection, stress, and trauma), and psychosocial factors (3, 4) Their core pathological manifestation is cerebral dysfunction, which leads to severe impairments in patients’ cognition, emotion, volition, and behavior.

Currently, the diagnosis of mental disorders relies primarily on clinical symptom assessment based on the criteria of the Diagnostic and Statistical Manual of Mental Disorders (DSM-5) or the International Classification of Diseases (ICD-11). This approach is highly dependent on clinicians’ experience and lacks objective and reliable biomarkers, resulting in subjective diagnosis, high heterogeneity, and often delayed early intervention (5, 6). In terms of treatment, pharmacotherapy with antipsychotics, antidepressants, and mood stabilizers, supplemented by psychotherapy and physical therapy, constitutes the current standard of care (7, 8). However, these therapies have significant limitations. For example, their efficacy is limited; they are poorly effective for a considerable subset of patients (e.g., those with treatment-resistant depression or schizophrenia) or alleviate only some symptoms. Second, notable side effects—such as metabolic syndrome (weight gain, diabetes), cardiovascular problems, and extrapyramidal symptoms—severely affect patient adherence and quality of life. Finally, these treatments address symptoms rather than causes; existing drugs mainly act on neurotransmitter systems (e.g., dopamine, serotonin) and fail to fundamentally reverse or halt the neurodegenerative process of the diseases.

Therefore, there is an urgent need to elucidate the pathogenesis of mental disorders from a novel perspective and to develop etiology-based, precise diagnostic and therapeutic strategies.

In recent years, the rise of neuroimmunology has provided a fresh perspective for understanding mental disorders. Accumulating evidence indicates that both immune cells within the central nervous system (CNS)—such as microglia and astrocytes—and peripheral immune cells—such as T cells and monocytes—undergo significant functional and phenotypic changes in mental disorders (9, 10). Concurrently, immunometabolism—an interdisciplinary field studying how metabolic pathways in immune cells regulate their function and fate—has revealed that changes in immune cell activity are always accompanied by profound metabolic reprogramming. For example, activated microglia shift from oxidative phosphorylation (OXPHOS) to aerobic glycolysis, resembling the Warburg effect in tumor cells (11, 12).

Although metabolic disturbances and immune abnormalities in mental disorders have been reported separately, a key scientific question remains systematically unexplored: how do alterations in metabolic pathways of central and peripheral immune cells specifically participate in the onset and progression of mental disorders? How are these cellular-level metabolic changes linked to patients’ systemic metabolic abnormalities (e.g., disturbances in glucose, lipid, and amino acid metabolism), and ultimately affect CNS function?

The literature for this narrative review was identified through systematic searches of PubMed, Web of Science, and Google Scholar up to December 2025, using combinations of keywords related to immunometabolism, neuroimmunology, metabolic pathways, and mental disorders. Given the breadth of the neuroimmunometabolism field and the narrative nature of this synthesis, we prioritized peer-reviewed original research, systematic reviews, and authoritative reviews published between 2000 and 2025, with emphasis on studies from 2015–2025. Reference lists of included articles were hand-searched to identify additional relevant studies.

While the neuroimmune metabolome encompasses a vast array of metabolites—including nucleotides, short-chain fatty acids, TCA cycle intermediates, and microbial derivatives—this review focuses on glucose, lipid, and amino acid (particularly glutamate and tryptophan) metabolism for three reasons (13, 14). First, these pathways represent the most extensively characterized and replicated findings across schizophrenia, AD/PD, and MDD (15). Second, they exhibit well-defined mechanistic links to both immune function and neuronal activity, with established molecular connections to neuroinflammatory responses (16). Third, these are the pathways for which therapeutic strategies targeting immunometabolism have shown preclinical promise, such as hexokinase 2 inhibition, ketogenic diet interventions, and IDO pathway modulators (17–19). A more comprehensive discussion of emerging metabolites, including SCFAs and microbial derivatives, is beyond the scope of this review but represents an important direction for future research (14).

This review aims to systematically integrate current evidence on metabolic pathway alterations (covering glucose, lipid, amino acid, and energy metabolism) in major central and peripheral immune cell populations—including microglia, astrocytes, T cells, B cells, natural killer (NK) cells, monocytes/macrophages, and neutrophils—in mental disorders, with a particular focus on schizophrenia, AD, PD, and depression. We further analyze how these metabolic changes reciprocally drive immune cell dysfunction, forming a vicious cycle that ultimately exacerbates neuroinflammation, neurotoxicity, and cognitive impairment. Importantly, we extend our analysis to therapeutic implications by critically reviewing emerging strategies that target immunometabolic reprogramming—including pharmacological interventions (e.g., hexokinase 2 modulators, kynurenine pathway inhibitors, CD38 checkpoint blockade, pioglitazone) and lifestyle approaches (e.g., ketogenic diet, exercise)—while also addressing the challenges and limitations that impede clinical translation, such as patient heterogeneity, context-dependent effects, and safety concerns. Finally, we identify gaps and future directions in this field, aiming to provide a theoretical foundation and new ideas for the precise diagnosis and mechanism-based treatment of mental disorders.

## 2 Metabolic alterations in major mental disorders

Metabolomics, the study of metabolic product types, quantities, and patterns of change following biological perturbations (such as genetic or environmental shifts), is critical for understanding mental disorders. Investigating metabolite variations in mental disorders is crucial for revealing disease mechanisms, improving diagnosis, and developing therapeutic approaches. Accumulating evidence indicates that carbohydrate (glucose) metabolism, lipid metabolism, amino acid metabolism, and energy metabolism are profoundly dysregulated in various mental disorders. However, the metabolic profiles differ significantly among disorders. To provide a clear and comparative view, this section presents the observed metabolic changes separately for schizophrenia, neurodegenerative diseases (Alzheimer’s disease, AD; Parkinson’s disease, PD), and major depressive disorder (MDD).

### 2.1 Schizophrenia

Patients with schizophrenia exhibit marked abnormalities in glucose metabolism. Studies reveal that individuals experiencing their first episode of psychosis (FEP) have significantly higher body glucose levels compared with healthy controls (20–24). However, at the cellular level, findings predominantly indicate a complex disturbance of glucose metabolism rather than a simple unidirectional decrease. For instance, several key glycolytic enzymes—including pyruvate dehydrogenase complex, triosephosphate isomerase, glyceraldehyde-3-phosphate dehydrogenase, phosphoglycerate kinase 1, and phosphoglycerate mutase—are elevated in the brain tissue of schizophrenia patients (25–29). Moreover, using targeted metabolomics in peripheral blood mononuclear cells (PBMCs), Liu et al. reported that the majority (84.6%) of glucose metabolites were significantly disturbed in schizophrenia subjects, with a particularly marked increase in ribose 5-phosphate, a key intermediate of the pentose phosphate pathway (PPP) (15). These observations indicate that glucose metabolism dysregulation in schizophrenia involves both altered glycolytic flux and reprogramming of the PPP. This complex disturbance is further supported by sustained expression changes in PPP-related molecules such as phosphoglycerate kinase 1, aldolase C, and glucose-6-phosphate dehydrogenase (28, 30).

Lipid metabolism is also markedly altered in schizophrenia. First-episode psychosis (FEP) patients exhibit elevated triglyceride levels (20), and genes associated with fatty acid metabolism and lipid synthesis are significantly downregulated (20, 31, 32). Approximately 40% of schizophrenia patients display abnormal lipid profiles (33). Importantly, the alterations in fatty acids are bidirectional and complex. While some studies report decreased levels of certain polyunsaturated fatty acids (PUFAs) (29), others have demonstrated significant increases in monounsaturated fatty acids (MUFAs) and ω-6 PUFAs in the serum of schizophrenia patients compared to healthy controls, accompanied by enhanced desaturation from saturated fatty acids to MUFAs and upregulated β-oxidation (34). Similar increases in MUFAs and PUFAs have been observed in the cholesteryl ester fraction of the prefrontal cortex in postmortem schizophrenia brains (35). A recent systematic review of lipidomics studies in schizophrenia spectrum disorders confirmed that fatty acyls—including various fatty acids—are dysregulated in patients, often in a direction that varies depending on antipsychotic treatment status (36). These findings indicate that schizophrenia involves global reprogramming of lipid metabolism rather than a simple unidirectional increase or decrease in fatty acid levels.

Amino acid metabolism disturbances are prominent. Schizophrenia patients show elevated levels of cysteine, GABA, glutamine, and carnitine, and decreased levels of arginine, ornithine, threonine, valine, tryptophan, methylcysteine, and hypoxanthine (37). Increased glutamate levels are observed in the thalamus, basal ganglia, and medial temporal lobe cortex (38). Elevated tryptophan metabolites (xanthurenic acid, kynurenine, quinolinic acid) in cerebrospinal fluid (CSF), brain tissue, or blood are common (39–45).

Energy metabolism is also affected. Early-stage schizophrenia research highlights activation of anaerobic glycolysis and the TCA cycle (15), with mitochondrial dysfunction implicated as a core feature (46).

### 2.2 Neurodegenerative diseases: Alzheimer’s disease and Parkinson’s disease

Reduced brain glucose metabolism is a hallmark of AD, PD, and other neurodegenerative diseases (47, 48). AD patients exhibit metabolic stress with impaired blood-brain glucose transfer and reduced glucose availability early in the disease (49, 50), correlating with downregulation of glucose transport proteins (GLUTs) (51).

Lipid metabolism alterations are evident in AD. Peripheral blood mononuclear cells from AD patients show significantly elevated neutral lipid concentrations (52) and an imbalance between free cholesterol and cholesterol esters (53, 54).

Amino acid metabolism changes include elevated glutamate and tryptophan catabolites. In AD mouse models, microglia show significant upregulation of glutamine, tyrosine, and threonine (55). PD patients also exhibit increased neurotoxic quinolinic acid levels.

Energy metabolism defects are profound. AD patients demonstrate impaired oxidative phosphorylation and mitochondrial dysfunction (56, 57). PD patients show similar mitochondrial abnormalities. Aging, a major risk factor for neurodegeneration, is associated with the emergence of lipid droplet-accumulating microglia (LDAM) characterized by upregulation of lipogenesis, TCA cycle, and fatty acid oxidation genes, alongside downregulated lipid degradation enzymes (58).

### 2.3 Major depressive disorder and other depressive conditions

Glucose metabolism is reduced in MDD. Patients exhibit decreased glucose metabolism in the prefrontal cortex (PFC) (59), and depression mouse models show reduced glucose oxidation in the PFC (60).

Lipid metabolism abnormalities include reduced cholesterol levels. Individuals with depressive symptoms (61, 62) or MDD (63, 64) frequently display decreased serum cholesterol.

Amino acid metabolism is centrally involved in the pathogenesis of MDD. Beyond merely representing downstream metabolic consequences of disease states, disruptions in amino acid metabolism—particularly the tryptophan–kynurenine pathway—actively contribute to the onset and progression of depression (25, 26). Compelling evidence indicates that the kynurenine pathway, activated by pro-inflammatory cytokines and stress-related signals, acts as a critical molecular bridge connecting peripheral and central inflammation to glutamatergic dysfunction in MDD (65). Under inflammatory conditions, the rate-limiting enzyme indoleamine 2,3-dioxygenase (IDO) is induced by pro-inflammatory cytokines (e.g., IFN-γ, TNF-α), converting tryptophan to kynurenine and shifting tryptophan metabolism away from serotonin synthesis toward the production of neuroactive kynurenine metabolites (66). Among these, quinolinic acid acts as an N-methyl-D-aspartate (NMDA) receptor agonist that drives excitotoxicity and neuroinflammation, whereas kynurenic acid is an NMDA receptor antagonist with neuroprotective and anti-inflammatory properties (67, 68). Patients with MDD exhibit kynurenine pathway dysregulation, often marked by an imbalance favoring neurotoxic quinolinic acid over neuroprotective kynurenic acid (65) This neurotoxic shift, together with an accompanying decrease in tryptophan availability for serotonin synthesis, has been implicated in the pathophysiology of MDD and may serve as a potential biomarker for disease severity and treatment response (69).

In line with this framework, clinical studies have reported elevated serum glutamate levels in MDD patients (70, 71), while the direction of change in glutamine levels varies. Conversely, in depression model mice subjected to chronic unpredictable mild stress (CUMS), glutamate and glutamine levels are significantly reduced (60). Depressed patients exhibit reduced circulating kynurenine (72), with some studies also reporting decreased quinolinic acid, particularly in the hippocampus (73). However, elevated quinolinic acid levels have been reported in other brain regions and in subgroups of MDD patients, highlighting the importance of region-specific and patient-subgroup analyses (68). The complexity of these findings likely reflects differences in disease stage, inflammatory status, and brain region examined, underscoring the need for further research into the role of the kynurenine pathway as a driver—rather than merely a correlate—of MDD pathogenesis.

Energy metabolism is impaired in MDD. Patients show reduced energy metabolism in the PFC, insula, and basal ganglia, including decreased total nucleoside triphosphates (NTP), β-nucleoside triphosphates, and high-energy phosphates (74–76). CUMS mice display reduced TCA cycle flux in astrocytes and decreased metabolic activity (60).

**Figure 1:**
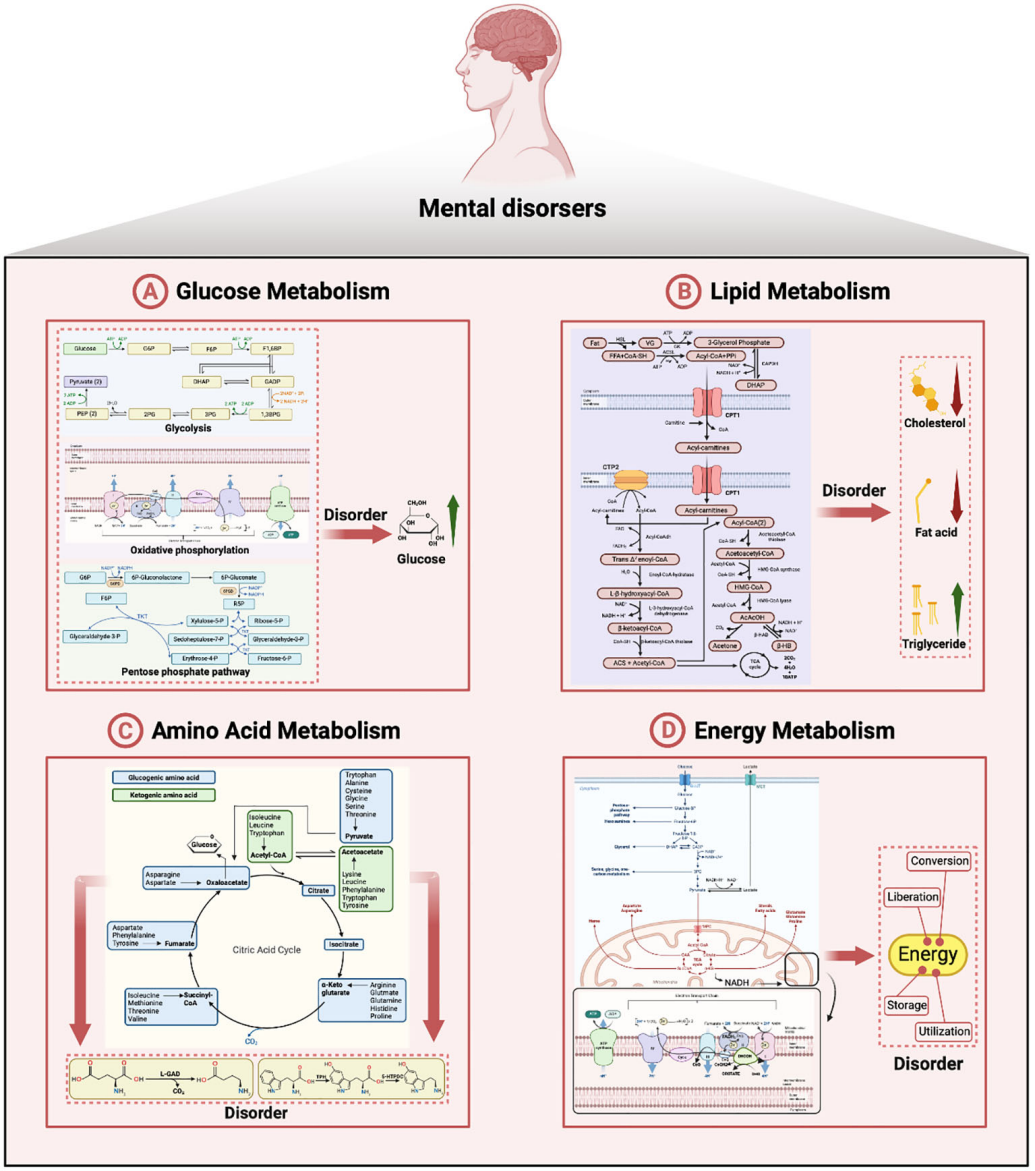
Overview of major metabolic pathway alterations in mental disorders: common themes and disease-specific variations. Created with BioRender.com. The image summarizes the most frequently reported changes across schizophrenia (SCZ), Alzheimer’s disease (AD), Parkinson‘s disease (PD), and major depressive disorder (MDD). However, the specific patterns and direction of change vary substantially by disease type, stage, and brain region. **(A)** Carbohydrate metabolism: Downregulated glucose metabolism and elevated peripheral glucose levels are most consistently reported in SCZ. In contrast, AD and MDD are associated with reduced glucose uptake and utilization in specific brain regions (e.g., prefrontal cortex), while PD shows regional hypometabolism in basal ganglia. **(B)** Lipid metabolism: SCZ features elevated triglycerides together with increased monounsaturated and ω-6 polyunsaturated fatty acids. MDD is characterized by reduced serum cholesterol. AD and aging show lipid droplet accumulation in microglia. **(C)** Amino acid metabolism: Elevated glutamate and tryptophan catabolites (e.g., kynurenine, quinolinic acid) are observed across all disorders, but with disease-specific patterns: SCZ shows increased kynurenic acid; MDD displays variable quinolinic acid levels depending on brain region and inflammatory state; AD/PD exhibit enhanced neurotoxic quinolinic acid. **(D)** Energy metabolism: Impaired oxidative phosphorylation and mitochondrial dysfunction are common to all disorders, but affected brain regions differ: frontal cortex in SCZ and MDD, hippocampus and cortex in AD, substantia nigra in PD.

In summary, while all four major metabolic pathways—glucose, lipid, amino acid, and energy metabolism—are altered across schizophrenia, neurodegenerative diseases (AD, PD), and major depressive disorder, the specific patterns differ substantially between disorders. Schizophrenia is characterized by hyperglycemia, complex lipid disturbances (including elevated triglycerides and altered fatty acid profiles), and increased glutamate/kynurenine levels. Neurodegenerative diseases feature reduced brain glucose uptake, lipid droplet accumulation in glial cells, and progressive mitochondrial dysfunction. Depression is associated with regional hypoglycemia (prefrontal cortex), low serum cholesterol, reduced TCA cycle activity, and disruptions in tryptophan–kynurenine metabolism that actively contribute to glutamatergic dysfunction and neuroinflammation. These distinct metabolic signatures suggest that targeting metabolism for diagnostic or therapeutic purposes may require disorder-specific approaches. **Figure 1** provides a visual summary of the major pathway-level changes, highlighting both common themes and disease-specific variations.

## 3 The immune landscape of mental disorders

To facilitate cross-disease comparison, this section describes the involvement of central and peripheral immune cells in schizophrenia (SCZ), neurodegenerative diseases (Alzheimer’s disease, AD; Parkinson’s disease, PD; and aging), and major depressive disorder (MDD). Findings are presented within each cell type but are explicitly labeled by disease, allowing the reader to appreciate both common and distinct immune alterations.

### 3.1 Central immune cell function in mental disorders

#### 3.1.1 The role of microglia in mental disorders

The central nervous system (CNS) is home to various glial cells, including microglia, astrocytes, and oligodendrocytes. Among these, microglia serve as the brain parenchyma’s resident innate immune cells, engaging directly with neurons and other glial types. Microglia actively sense and respond to a diverse array of pathophysiological signals—including pro-inflammatory cytokines, stress-associated molecular patterns, and disease-specific protein aggregates—through a broad repertoire of surface and intracellular receptors. Emerging evidence strongly indicates that microglial activation is not merely a consequence of disease states but rather contributes directly to the pathophysiology, and in some cases the pathogenesis, of mental disorders by modulating synaptic function, neuroinflammatory cascades, and neurotransmitter homeostasis (77–80).

##### 3.1.1.1 Upstream triggers of microglial activation in mental disorders

The activation of microglia in mental disorders is driven by various upstream factors, which differ across disease types and pathophysiological contexts.

In schizophrenia, peripheral and central inflammatory signals constitute the primary drivers of microglial activation. Elevated levels of pro-inflammatory cytokines—particularly IL-6, TNF-α, and IL-1β—are consistently observed in the peripheral blood and cerebrospinal fluid (CSF) of schizophrenia patients (81). These peripheral immune signals can activate microglia via humoral pathways through circumventricular organs and active transport mechanisms across the blood-brain barrier (BBB). Additionally, genetic susceptibility and environmental stressors (e.g., prenatal infection or psychosocial stress) may prime microglia, lowering their activation threshold and promoting exaggerated responses to subsequent immune challenges (82). These findings collectively indicate that microglial activation in schizophrenia is primarily driven by systemic and central inflammatory signals.

In neurodegenerative diseases such as Alzheimer’s disease (AD) and Parkinson’s disease (PD), the primary triggers of microglial activation are disease-specific protein aggregates. In AD, amyloid-β (Aβ) plaques and tau neurofibrillary tangles act as damage-associated molecular patterns (DAMPs), engaging pattern recognition receptors (PRRs) such as Toll-like receptors (TLRs) expressed on microglia (83). Microglial activation is initiated through PRR ligation by neurodegeneration-associated proteins (e.g., tau and Aβ), leading to NLRP3 inflammasome activation and subsequent release of IL-1β and IL-18 (84). In PD, α-synuclein (αSyn) oligomers similarly trigger microglial activation, driving an early neuroinflammatory phase characterized by the expansion of a high-SNCA-expressing microglial state before Lewy body formation (85). Importantly, microglia-driven inflammation alone has been shown to be sufficient to trigger the full range of pathological features in tauopathies and synucleinopathies (86).

In depression and stress-related disorders, chronic psychological stress serves as a key upstream driver of microglial activation. Prolonged exposure to stress elevates circulating glucocorticoid levels and promotes peripheral monocyte mobilization, which in turn activates microglia via systemic inflammatory signals (82). At the molecular level, chronic stress upregulates the mitochondrial protein CDGSH iron-sulfur domain 1 (CISD1) in microglia, promoting NADH oxidation to NAD^+^, accelerating glycolysis, and driving inflammatory activation (87). This stress-induced microglial metabolic reprogramming has been causally linked to the development of depressive-like behavior in animal models (87).

###### 3.1.1.1.1 Linking disease-specific metabolic milieus to microglial functional outcomes

The unique metabolic environment of each disease directly shapes how microglia respond and transition between functional states. In schizophrenia, patients exhibit peripheral hyperglycemia and insulin resistance alongside elevated circulating pro-inflammatory cytokines (20–24). This hyperglycemic milieu provides increased glucose substrate availability for microglia, which upon activation shift toward aerobic glycolysis and upregulate the pentose phosphate pathway (15). The resulting glycolytic flux sustains the production of pro-inflammatory cytokines (IL-1β, TNF-α, IL-6) and facilitates the conversion of tryptophan toward neurotoxic kynurenine metabolites, thereby amplifying the inflammatory response in a feed-forward loop. Importantly, chronic exposure to elevated glucose may also induce a state of metabolic tolerance in microglia, reducing their responsiveness to subsequent stimuli and contributing to the persistent low-grade neuroinflammation observed in schizophrenia.

In Alzheimer’s disease, the primary metabolic drivers are distinct. Amyloid-β (Aβ) plaques and tau neurofibrillary tangles act as damage-associated molecular patterns (DAMPs), engaging Toll-like receptors (TLRs) on microglia (88). This engagement triggers not only classical inflammatory signaling but also a profound metabolic shift toward glycolysis, mediated in part by hexokinase 2 (HK2) upregulation (89). Activated microglia in early AD exhibit increased glucose uptake and lactate production, supporting rapid immune responses. However, with chronic exposure to Aβ, microglia transition into a dysfunctional senescent state characterized by impaired glucose uptake, mitochondrial dysfunction, reduced phagocytic capacity, and accumulation of lipid droplets (56, 58, 90). The presence of APOE4, a major genetic risk factor for late-onset AD, exacerbates this dysfunction by impairing microglial lipid metabolism, leading to cholesterol accumulation that inhibits neuronal firing and promotes disease progression (91). Thus, in AD, microglial metabolic reprogramming evolves from an acute protective phase to a chronic maladaptive phase that drives neurodegeneration.

In Parkinson’s disease, α-synuclein (αSyn) oligomers similarly drive microglial activation through distinct metabolic signatures. αSyn triggers an early neuroinflammatory phase characterized by the expansion of a high-SNCA-expressing microglial state before Lewy body formation (85). Microglia in PD shift toward glycolysis and exhibit impaired mitochondrial oxidative phosphorylation, leading to the accumulation of reactive oxygen species that further damage dopaminergic neurons.

In depression and stress-related disorders, the metabolic drivers are fundamentally different from those in neurodegenerative diseases. Chronic psychological stress upregulates the mitochondrial protein CDGSH iron-sulfur domain 1 (CISD1) in microglia, promoting NADH oxidation to NAD^+^ and accelerating glycolysis (87). This stress-induced metabolic reprogramming has been causally linked to depressive-like behavior in animal models (87). Furthermore, chronic stress induces microglial atrophy and loss in the hippocampus of depressed patients (92), contrasting with the reactive microgliosis seen in schizophrenia and AD. The direction of change—whether microglia are hyperactivated, senescent, or lost—is thus disease-dependent and determined by the underlying metabolic trigger.

##### 3.1.1.2 Microglial phenotypic alterations across mental disorders

In line with the upstream triggers described above, extensive studies have documented consistent alterations in microglial number, activation state, and polarization across mental disorders.

In schizophrenia, microglial activation is well documented (77, 78). Analyses of patient brain tissues reveal an increase in both total and activated microglial populations (93, 94). Autopsies of schizophrenia patients who succumbed during acute psychotic episodes also demonstrate increased microglial density (95). Furthermore, an imbalance in M1/M2 polarization of microglia is implicated in the disease’s pathogenesis (96). Upon activation, microglia can differentiate into M1 (pro-inflammatory) or M2 (anti-inflammatory) states. Schizophrenia’s recurrence may be linked to pro-inflammatory M1 polarization, whereas its alleviation is tied to anti-inflammatory M2 polarization, which curtails M1 microglial activity and upholds tissue homeostasis (97).

In neurodegenerative diseases (AD, PD, and aging), activated microglia are prevalent in AD (98). In early stages, an M2 phenotype predominates, helping to inhibit β-amyloid accumulation and neurofibrillary tangles (99). In advanced stages, prolonged exposure to pathogens such as β-amyloid prompts microglia to enter a dysfunctional senescent state with reduced phagocytic capabilities and a tolerant condition marked by a decrease in pro-inflammatory compound release (100). AD mouse models reveal a transition of microglia from a homeostatic to disease-associated phenotype as the disease advances (101), encompassing disease-associated microglia (DAM), phagocytically active microglia (PAM), microglial neurodegenerative phenotype (MGnD), and other types (102–104). In 5xFAD and aged mice, acute exposure to β-amyloid enhances microglial phagocytosis and chemotaxis (105, 106). Increased microglial activation is also observed in the brains of PD patients (107), with activated microglia distributed across several brain regions, including the striatum (108, 109). Aging contributes to morphological changes in microglia in both mouse and human brains (58), pushing microglia toward a pro-inflammatory state (110).

In depression, depressive behaviors correlate with loss of microglia in the hippocampus (92), and post-mortem examinations of depression patients show activated microglia in the brain (98). Major depressive disorder (MDD) patients exhibit a significant increase in activated microglia within the prefrontal cortex, anterior cingulate cortex, and insula (111), with evidence of activated microglia in the posterior cingulate gyrus material of MDD patients after death (112). Overactivation of neural circuits in MDD patients triggers M1 polarization of microglia (97). These findings highlight that mental disorders are associated with profound alterations in microglial activity in the CNS.

##### 3.1.1.3 Mechanisms linking microglial activation to disease pathology

The phenotypic alterations described above translate into disease-relevant pathology through multiple molecular mechanisms.

First, activated microglia disrupt the kynurenine pathway of tryptophan metabolism. In schizophrenia, microglia emit neuroimmune signals that stimulate the kynurenine pathway, shifting the balance between quinolinic acid (QUIN) and kynurenic acid (KYNA). This dysregulation, characterized by altered QUIN/KYNA ratios, leads to glutamate metabolism disturbances and exacerbates psychotic symptoms (113–119). Microglial activation also facilitates the conversion of glutamate to glutamine, ultimately contributing to NMDA receptor antagonism and neuronal apoptosis (113).

Second, microglial metabolic reprogramming—particularly the switch from oxidative phosphorylation to glycolysis—drives the production of pro-inflammatory cytokines and neurotoxic molecules. In AD, chronic exposure to Aβ and tau proteins impairs microglial glucose uptake and mitochondrial function, leading to metabolic dysfunction that perpetuates neuroinflammation (56, 90, 105). APOE4, the strongest genetic risk factor for late-onset AD, disrupts microglial lipid metabolism, resulting in cholesterol accumulation that inhibits neuronal firing and reduces excitability, thereby promoting AD progression (91). Glycolytic enzymes such as HK2 are notably upregulated in AD microglia; HK2 knockout restores microglial phagocytic function and improves cognition in AD mice by mobilizing intracellular lipid metabolism (119).

Third, microglial-derived inflammatory mediators directly affect neuronal function. Pro-inflammatory cytokines (IL-1β, TNF-α) released from activated microglia impair long-term potentiation and synaptic plasticity (120). In stress-induced depression models, microglial activation promotes the recruitment of IL-1β-producing monocytes to brain endothelium, causing anxiety-like behavior (121).

Collectively, these mechanisms establish microglial activation as a central hub connecting upstream triggers (inflammation, protein aggregates, chronic stress) to downstream neuropathology (glutamate dysregulation, metabolic dysfunction, synaptic impairment) across diverse mental disorders.

### 3.2 The role of peripheral immune cells in mental disorders

The influence of the peripheral immune system on the central nervous system (CNS) has gained recognition over recent years, highlighting various communication pathways between these two systems. This “communication” suggests that peripheral immune cells are integral to the development of mental disorders. Peripheral immune cells, including T lymphocytes, B lymphocytes, NK cells, monocytes/macrophages, and neutrophils, each play distinct roles in mental disorders.

#### 3.2.1 The role of T lymphocytes in mental disorders

The involvement of the peripheral immune system in mental disorders highlights the complex interplay between the body’s immune response and the CNS. Notably, T lymphocytes play a significant role in this relationship. Patients with schizophrenia exhibit an increase in CD3^+^ and CD4^+^ T cells upon admission, particularly noted by a rise in CD4^+^ cells (122). Acute psychotic episodes in First Episode Psychosis (FEP) patients also see an increased percentage of CD4^+^ T cells in the blood (123), alongside an increased density of T cells in mood disorder patients (124). Conversely, some studies report a reduction in Th cells in schizophrenia (125), significantly correlated with a decrease in CD4^+^ cell percentage and the CD4^+^/CD8^+^ ratio (125). Moreover, an increase in CD36 expression on CD4^+^ and CD4^-^CD8^-^ T cells in schizophrenia (126) suggests these cells’ involvement in CNS processes, particularly in the regulation of astrocyte activation (127). The differentiation into Th cell subtypes, such as Th1, Th2, Th17, and Tregs (regulatory T cells), further illustrates the complexity of T lymphocyte involvement. Schizophrenia patients, for example, display a reduced overall number of circulating Tregs (128), though another study found Treg abundance increased while their activity is decreased (129). Pro-inflammatory Th17 cells are also elevated in schizophrenia (128), and an imbalance or shift toward Th2 cells is associated with the condition (130). Neurodegenerative diseases, including Mild Cognitive Impairment (MCI) and Alzheimer’s Disease (AD), show alterations in T lymphocytes as well. An increase in CD8^+^ T effector memory CD45RA^+^ (TEMRA) cells is observed in both peripheral blood and cerebrospinal fluid (CSF) of these patients (131). Post-mortem examinations of AD patients reveal T cell proliferation in CSF, meninges, and the hippocampus (131, 132), suggesting T cell infiltration into the CNS is closely linked to disease onset. In addition, the occurrence of aging is related to the reduction in the number and function of CD4^+^T and CD8^+^T cells (133). Although aging can lead to a decrease in the initial number of T cells in the circulation, the number of Treg cells increases (134). Experiments on mice have demonstrated that acute psychological stress can increase T cell migration to the brain (135). Inflammatory depression is characterized by an uptick in CD4^+^ T cells (136), and stress and depression are known to dampen the response of T cells, especially T regulatory cells (137, 138). These findings highlight the significant role of T lymphocytes in the onset and progression of mental disorders.

#### 3.2.2 The role of other peripheral immune cells in mental disorders

B lymphocytes have also shown variable responses in mental disorders. In schizophrenia, there’s a reported decrease in B lymphocytes (125), significantly linked to a reduced percentage of these cells (125). Yet, increases in B cell density have been observed in some patients with schizophrenia and mood disorders (124). In AD, a neurodegenerative condition, there’s an increased frequency of IgG-producing B cells in the serum, despite a decrease in the total number of B cells (139, 140). However, the accumulation of B cells can be found in an elderly mouse model (141). The number of circulating B lymphocytes in patients suffering from depression has been observed to increase (142, 143), whereas the count of regulatory B lymphocytes responsible for producing IL-10 has declined (142, 144), suggesting a potential correlation between depression and an expanded yet dysfunctional peripheral B cell compartment.

Then, Natural Killer (NK) cells exhibit changes across various mental disorders. FEP patients experience an increase in NK cell percentage during acute psychotic episodes (123), with genes related to NK cell activation being upregulated (20). Studies on schizophrenia have consistently found an increase in NK cell numbers (125, 145, 146), significantly associated with an uptick in both the absolute number and percentage of NK cells (125). Furthermore, NK cells derived from peripheral blood in AD have been shown to migrate to the CNS, implicating their involvement in AD development (147, 148). In addition, aging can lead to an increase in the total number of NK cells (134). Conversely, a decrease in NK cell numbers has been reported in patients with Major Depressive Disorder (MDD) (149), suggesting a disease-specific impact on NK cell levels.

And, the role of monocytes/macrophages in mental disorders has been increasingly recognized. In schizophrenia, there is a notable increase in peripheral blood monocyte counts (150), with similar elevations observed in patients with chronic schizophrenia and first-episode psychosis (151). Monocytes and macrophages are also found to accumulate in the cerebrospinal fluid (CSF) of schizophrenia patients (152), and recent studies have identified their infiltration into the prefrontal cortex in brains of individuals with schizophrenia, alongside increased peripheral inflammation (153). This accumulation in both peripheral blood and brain tissue of schizophrenia patients highlights the significant involvement of monocytes/macrophages in the disease. Furthermore, in AD, peripheral blood-derived monocytes/macrophages can be recruited to the CNS to contribute to AD development (147, 148). Throughout AD progression, there’s a decrease in classical monocytes, while non-classical and intermediate monocytes increase alongside rising Aβ load (154), indicating a phenotypic shift from anti-inflammatory to pro-inflammatory (154, 155). Observations in AD patients also reveal an increase in the M1 macrophage subgroup and a decrease in the M2 phenotype, particularly in the M2b subgroup, correlating with cognitive impairment (156). These findings illustrate the close relationship between the subtype and number of monocytes/macrophages and AD. Research in Parkinson’s Disease (PD) has shown improved phagocytic function in monocytes (157), with disease occurrence linked to increased monocyte brain infiltration (158). Chronic stress leads to elevated circulating monocyte levels (159) and increased blood-brain barrier (BBB) permeability, facilitating monocyte transport to the brain (160, 161). The aging mouse model showed a significant decrease in the number of peritoneal monocytes/macrophages and enhanced inflammatory function (162). Increases in peripheral blood monocytes have also been observed in patients with depression (136, 163, 164), with both blood samples from Major Depressive Disorder (MDD) patients and depression mouse models showing elevated monocyte counts (165). Increased macrophage recruitment in the posterior cingulate gyrus of MDD patients post-mortem has been documented (112). These comprehensive findings demonstrate the critical connection between monocyte/macrophage activity and the onset and progression of mental disorders.

Lastly, neutrophils have also shown significant changes in patients with mental disorders. In chronic schizophrenia and first-episode psychosis, neutrophil levels in the blood are elevated (151). Schizophrenia patients experience compromised neutrophil chemotaxis, phagocytosis, and oxidative metabolism (125). In AD, the number of neutrophils significantly increases, particularly during periods of memory deficits (166). Peripheral blood-derived neutrophils can be recruited to the CNS, contributing to AD’s development (147, 148). Similarly, an increase in neutrophils has been observed in the peripheral blood of depression patients (136), with studies noting elevated neutrophil counts in both MDD patients and depression mouse models (165). The ratio of neutrophils to lymphocytes is also linked to depression severity (167, 168). These findings highlight the importance of changes in neutrophil activity in mental disorder progression.

Collectively, these findings emphasize that the alterations in peripheral cells, including changes in number, spatial distribution and activation status, involve the occurrence and development of mental diseases or symptoms, such as schizophrenia, neurodegenerative diseases and depression. See **Table 1** for details.

**Table 1:** Peripheral immune cell function and metabolic pathway in schizophrenia, neurodegenerative diseases and depression.

Disease type	Cell type	Immune cell function	Immune cell metabolism pathway
Schizophrenia	T Lymphocytes	Schizophrenia patients exhibit an increase in CD4^+^T cells, including increased percentage, density, and CD36 expression (87, 122–141, 143–222). And the Treg subtype shows a decrease in total numbers (128) or an increase in abundance (129)^]^; the Th17 subtype is significantly elevated (129); and there’s a Th1/Th2 imbalance skewed toward Th2 (130)^]^.	Amino Acid Metabolism: Tryptophan metabolism disorders are observed in CD4^+^T cell subtypes of schizophrenia patients (169, 170).
	B Lymphocytes	Schizophrenia patients show a decrease in B lymphocyte numbers, mainly in percentage (160); some reports also observe an increase in B lymphocyte density (124).	
	NK Cells	Schizophrenia patients show an increase in NK cell numbers (125, 145, 146), with both absolute numbers and percentages significantly increased (125).	
	Monocytes/Macrophages	Schizophrenia patients show a significant increase in peripheral blood monocytes (150, 151), and accumulation of monocyte-macrophages in brain tissue (152)^]^.	Glucose Metabolism: Schizophrenia patients exhibit abnormalities in peripheral blood mononuclear cell glucose metabolism, with elevated glucose metabolism enzyme activity (25–29). Additionally, pentose phosphate pathway disorders and significantly increased ribose 5-phosphate are observed (15).
	Neutrophils	Schizophrenia patients exhibit elevated blood neutrophil levels (151), while neutrophil chemotaxis, phagocytosis, and oxidative metabolism are impaired (125).	
Neurodegenerative diseases [Alzheimer’s disease (AD), Parkinson’s disease (PD), and aging]	T Lymphocytes	(1) AD patients show T cell proliferation in cerebrospinal fluid, meninges, and hippocampus (131, 132), with increased CD8^+^ effector memory T cells observed in both peripheral blood and brain (131). (2) Aging is associated with a reduction in CD4^+^ and CD8^+^T cell numbers and function (133). And aging leads to a decrease in naive T cells in circulation, while an increase in Treg cell numbers (134).	Glucose Metabolism: Aging reduces glycolysis in CD4^+^T cells (171). Activated T cells during aging show reduced levels of metabolites in glycolysis and the pentose phosphate pathway (171). Energy Metabolism: Aging weakens the ability of CD4^+^T cells to induce one-carbon metabolism (172). T cell activation during aging leads to reduced levels of metabolites in the TCA cycle (171).
	B Lymphocytes	(1) AD patients show a decrease in total B lymphocytes, but an increase in the frequency of IgG-producing B cells in serum (139, 140). (2) Aged mouse models show accumulation of B cells (141).	Glucose Metabolism: Aging reduces glycolysis in B cells (173). Energy Metabolism: Aging causes mitochondrial energy production defects in B cells (173).
	NK Cells	(1) AD patients show NK cells from peripheral blood are recruited to the CNS (147, 148). (2) Aging leads to an increase in total NK cell numbers (134).	Lipid Metabolism: NK cells from elderly subjects show significantly reduced production of inositol triphosphate (IP3) (174). Amino Acid Metabolism: NK lymphocytes show increased indoleamine 2,3-dioxygenase (IDO) activity in some neurodegenerative diseases (175).
	Monocytes/Macrophages	(1) AD patients show peripheral blood monocytes/macrophages can be recruited to the CNS (176, 177). During AD progression, classical monocytes decrease, while non-classical and intermediate monocytes increase with increasing Aβ load (154). An increase in M1 macrophages and a decrease in M2 phenotype are observed in AD patients (156). (2) Parkinson’s disease (PD) patients show improved phagocytic function of mature monocytes (157) and increased monocyte brain infiltration (158). (3) Aged mouse models show a significant decrease in peritoneal monocyte/macrophage numbers with enhanced inflammatory function (162).	(1) AD Lipid Metabolism: Peripheral blood mononuclear cells in Alzheimer’s disease (AD) patients show significantly elevated neutral lipid concentrations (52), with an imbalance in free cholesterol and cholesterol esters (53, 54). (2) Aging Glucose Metabolism: Circulating monocytes/macrophages in aging patients are glucose-dependent, with significantly reduced glycolysis (178, 179). Energy Metabolism: Circulating monocytes/macrophages in aging patients show significantly reduced mitochondrial oxidative phosphorylation, ultimately leading to energy deficiency (178, 179).
	Neutrophils	AD patients show a significant increase in neutrophil numbers, with peripheral blood-derived neutrophils recruited to the CNS (147, 148).	
Depression	T Lymphocytes	Depression is characterized by an increase in CD4^+^T cells (136). Stress-induced anxiety-depression mouse models show T cell migration to the brain (135) and inhibition of Treg cells (137, 138).	Glucose Metabolism: Naive CD4^+^T cells in mouse anxiety models show significantly reduced glycolysis levels (180). 2. Energy Metabolism: Naive CD4^+^T cells in mouse anxiety models show significantly reduced oxidative phosphorylation (OXPHOS) levels (180).
	B Lymphocytes	Depression patients show an increase in circulating B lymphocyte numbers (142, 143), while inhibitory regulatory B lymphocytes decrease (142, 144).	
	NK Cells	Major Depressive Disorder (MDD) patients show a decrease in NK cell numbers (149).	
	Monocytes/Macrophages	Depression patients show an increase in peripheral blood monocyte numbers (136, 163, 164). MDD patients and depression mouse models also show increased peripheral blood monocyte numbers (165), with increased monocyte-macrophage recruitment in the brain of MDD patients (112).	Lipid Metabolism: Cholesterol metabolism-related genes are significantly upregulated in monocytes of MDD patients (181). Energy Metabolism: Monocytes in MDD patients show mitochondrial dysfunction (181).
	Neutrophils	Depression patients show an increase in peripheral blood neutrophils (136). MDD patients and depression mouse models also show elevated neutrophil numbers (165). The neutrophil-to-lymphocyte ratio correlates with the severity of depression (167, 168).	

## 4 Metabolic pathway changes in immune cells related to mental disorders

### 4.1 The metabolic pathway changes in central immune cells related to mental disorders

#### 4.1.1 The metabolic pathway changes in microglia related to mental disorders

Changes in microglial activity, including variations in their number, activation state, or type, inevitably affect microglial metabolism pathway. Upon activation or transition to an inflammatory state, microglial bioenergetic metabolism shifts from aerobic oxidative phosphorylation (OXPHOS) to anaerobic glycolysis, with ATP generation also relying on the pentose phosphate pathway (PPP). This shift indicates that in an inflammatory state, microglial energy metabolism undergoes significant changes, chiefly marked by increased glucose uptake and utilization to sustain cellular energy needs (66, 176, 177, 182–189). Further investigations reveal that M1 microglia increase glucose and glutamine oxidation while reducing mitochondrial fatty acid oxidation (FAO), utilizing glucose through the TCA cycle without alterations in the pentose phosphate pathway (190). In contrast, M2 microglia increase mitochondrial FAO and fatty acid synthesis. These results indicate that the activity and state of microglia can affect their metabolic pathways, mainly including glucose metabolism, lipid metabolism, amino acid metabolism and energy metabolism.

In the context of mental disorders, such as schizophrenia, alterations within the brain’s architecture provoke a response from microglia. Microglia emit neuroimmune signals that stimulate the metabolic cascade pertaining to canine uric acid, thereby disrupting the equilibrium between quinolinic acid (QUIN) and kynurenic acid (KYNA) levels. This shift entails a persistent decline in QUIN concentrations and a concomitant rise in KYNA, with QUIN’s diminished presence in the brain inhibiting the N-methyl-D-aspartate (NMDA) receptor. The interplay between these two factors culminates in glutamate metabolism dysregulation, exacerbating psychotic manifestations (113–119). Furthermore, the activation of microglia significantly impacts glutamate metabolism, facilitating its conversion into glutamine. This transformation ultimately results in NMDA receptor antagonism and neuronal apoptosis in schizophrenia patients, highlighting the intricate connection between microglia activity and amino acid metabolic pathways, particularly those involving glutamate (113). In addition, Alzheimer’s disease also involves multiple metabolic pathways of microglia. Early-stage AD is characterized by activated microglia with increased glucose consumption, whereas late-stage AD microglia experience metabolic reprogramming damage, decline in mitochondrial function, and impaired glucose uptake (56, 90). This suggests a connection between AD microglia dysregulation and glucose metabolism impairment (191). Increased glycolysis has also been noted in microglia from AD mouse models (192, 193), with acute β-amyloid exposure boosting microglial glycolysis in 5xFAD and aged mice, though chronic exposure leads to metabolic dysfunction (105, 106). Thus, in both AD patients and animal models, microglial glucose metabolism pathways undergo modifications; prolonged reduced glucose availability in AD prompts microglia to utilize alternative energy sources. Fatty acids (FA) released from lipid droplet degradation, are transported to mitochondria for fueling mitochondrial OXPHOS through fatty acid oxidation (FAO). Research identifies numerous FA-related genes expressed in microglia (223), suggesting that AD involves alterations in lipid metabolism pathways. Transcriptome sequencing of whole brain tissue from AD mouse models (expressing APOE3 or APOE4) reveals that APOE4 LPS-activated brain microglia exhibit increased activity in the amino acid metabolism pathway. Targeted metabolomics methods have pinpointed significant upregulations of glutamine, tyrosine, and threonine in APOE4 microglia (55), linking AD to changes in the amino acid metabolism pathway. Additionally, proteomics analyses of AD brains show that activated microglia are enriched in proteins associated with energy metabolism (56, 57). Long-term exposure to pathogens, such as β-amyloid and tau protein, results in mitochondrial toxicity and metabolic dysfunction in microglia (105), tying AD to energy metabolism dysregulation. Beyond AD, aging contributes to the accumulation of lipid droplets in microglia in both mouse and human brains, leading to the identification of lipid droplet-accumulating microglia (LDAM) (58). LDAMs are characterized by the upregulation of genes related to lipogenesis, the TCA cycle, and FAO, alongside increased ROS production, while key enzymes for lipid degradation are downregulated (58). This indicates that aging also influences lipid metabolism pathway dysregulation. Additionally, in MDD patients, elevated quinolinic acid levels are observed in microglia in the midbrain, hypothalamus, and superior vena cava, while with decreased levels in the hippocampus (73). These findings affirm the close connection between mental disorders and changes in microglial metabolic pathways, the details in **Table 2**.

**Table 2:** Central immune cell function and metabolic pathway in schizophrenia, neurodegenerative diseases and depression.

Disease type	Cell type	Immune cell function	Immune cell metabolism pathway
Schizophrenia	Microglia	Schizophrenia patients exhibit increased number, density, and activity of microglia, and M1/M2 polarization imbalance (77, 78, 93–97).	Amino acid metabolism pathway: Brain structural alterations in patients with schizophrenia lead to microglial activation, triggering the kynurenine pathway, resulting in an imbalance of quinolinic acid and kynurenic acid ratios, ultimately causing glutamate metabolism dysregulation and psychotic symptoms (113–119). Microglial activation modulates glutamate metabolism, facilitating the conversion of glutamate to glutamine (113).
	Astrocytes	Schizophrenia patients exhibit increased astrocyte numbers and activity (224, 225); Other studies report decreased astrocyte density and attenuated expression of the activity marker (226–235).	Amino acid metabolism pathway: Astrocyte activation modulates glutamate metabolism (113).
Neurodegenerative diseases[Alzheimer’s disease (AD), Parkinson’s disease (PD), and aging]	Microglia	(1) Alzheimer’s disease (AD) AD patients demonstrate early microglial activation, transitioning to a dysfunctional state in later stages (98–100); AD mouse models exhibit microglial phenotype transition from homeostatic to disease-associated, characterized by enhanced phagocytic activity (101–106). (2) Parkinson’s disease (PD) PD patients exhibit increased microglial activation (107–109). (3) Aging Aging induces morphological alterations in mouse and human brain microglia, shifting toward a proinflammatory state (58, 110).	(1) Alzheimer’s disease (AD) Glucose metabolism pathway: Early-stage AD patients exhibit increased glucose consumption, while late-stage patients demonstrate impaired glucose uptake (56, 90); AD mouse model microglia show upregulated glycolysis (233, 234). Lipid metabolism pathway: AD patient microglia upregulate FA-related genes, promoting fatty acid oxidation (FAO) transport to mitochondria for energy production (223). Amino acid metabolism pathway: AD mouse models reveal significant upregulation of glutamine, tyrosine, and threonine in microglia (55). Energy metabolism pathway: AD patient microglia exhibit enrichment in energy metabolism-related proteins (57, 89). (2) Aging Lipid metabolism pathway: Mouse and human brains display lipid droplet-accumulating microglia (LDAM), characterized by upregulation of genes associated with lipogenesis, TCA cycle, and FAO (58).
	Astrocytes	(1) Alzheimer’s disease (AD) Early-onset AD animal models exhibit increased astrocyte numbers in the posterior cortex; astrocyte activation is considered a risk factor for early-onset AD patients (236). Pathological astrocyte activation and proliferation are observed around Aβ plaques in AD patients (237). Astrocyte proliferation, including increased volume and thickened processes, is prevalent in AD (238). (2) Parkinson’s disease PD patients demonstrate activated astrocytes (239, 240).	Alzheimer’s disease Glucose metabolism pathway: AD patient astrocytes exhibit increased glucose flux through the pentose phosphate pathway (PPP), leading to enhanced gluconeogenesis and phospholipid and nucleotide biosynthesis (142). Lipid metabolism pathway: AD patient astrocytes display cholesterol accumulation (241–245). Energy metabolism pathway: AD patient astrocytes demonstrate impaired mitochondrial homeostasis and attenuated oxidative phosphorylation (241–245).
Depression	Microglia	(1) Stress-induced depressive-like behavior in mice is associated with microglial depletion (92). (2) Depression patients exhibit microglial activation (98). (3) Major Depressive Disorder (MDD) patients exhibit increased activated microglia (111, 112), triggering M1 polarization (97).	MDD Amino acid metabolism pathway: Elevated quinolinic acid levels are observed in microglia in the midbrain, hypothalamus, and superior vena cava of MDD patients, with decreased levels in the hippocampus (73).
	Astrocytes	(1) Depressive behavior is associated with astrocyte atrophy (246). (2) Chronic stress-induced animal depression models demonstrate decreased astrocyte density (247). (3) MDD patients exhibit reduced astrocyte density (248).	(1) Chronic stress-induced animal depression models Glucose metabolism pathway: Depression model exhibits reduced glucose oxidation rate in astrocytes (249). Amino acid metabolism pathway: Depression model demonstrates alterations in astrocyte glutamate and GABA metabolism cycles and neurotransmitter cycling (249). Energy metabolism pathway: Depression model shows attenuated TCA cycle flux in astrocytes (60). (2) Major Depressive Disorder (MDD) Amino acid metabolism pathway: MDD patients exhibit downregulation of astrocyte genes and proteins related to glutamate and GABA metabolism (250).

These metabolic alterations in microglia further drive disease progression. With changes in the metabolic network state observed in AD cell models and transgenic mouse brains, metabolic reprogramming and dysfunction can trigger microglial activation, leading to an overproduction of pro-inflammatory cytokines and neurotoxic molecules, thereby exacerbating neural inflammation and neurotoxicity in AD patients (194). Post-mortem proteomics analyses of AD patient brains have identified microglial glycolysis “switches” such as LDHB (lactate dehydrogenase), PKM (pyruvate kinase), and glyceraldehyde 3-phosphate dehydrogenase (GAPDH) potentially involved in AD pathogenesis (56), highlighting the influence of microglial glucose metabolism products on AD onset. Furthermore, APOE4, the most significant genetic factor linked to late-onset non-familial AD, disrupts microglial lipid metabolism, resulting in the accumulation of lipid molecules, particularly cholesterol. This accumulation affects specific potassium ion channels on neuronal cell membranes, inhibiting neuronal firing, reducing excitability, and promoting AD development (91). Lipid accumulation in microglia also leads to inflammation, regarded as a contributing factor to AD progression (91), signifying the impact of microglial lipid metabolism products on AD pathogenesis. Moreover, the glycolytic enzyme HK2 is notably upregulated in the microglia of AD patients and mice. Studies have shown that HK2 knockout in microglia significantly promotes microglial phagocytosis of amyloid, thereby improving cognitive function in AD mice (89), suggesting the influence of microglial glucose metabolism products on AD pathogenesis. Although HK2 knockout reduces microglial glucose utilization, it triggers an upregulation of the key lipid metabolism enzyme lipoprotein lipase (LPL), mobilizing intracellular lipid metabolism in microglia. This adjustment rapidly increases intracellular ATP levels, enhancing microglial phagocytic function. Additionally, HK2’s downstream metabolic products, glucose-6-phosphate (G-6-P) and fructose-6-phosphate (F-6-P), can regulate lipid metabolism via the pentose phosphate pathway, linking sugar-lipid metabolism to microglial functions such as phagocytosis (89). This further confirms the critical role of microglial metabolic pathways in disease progression.

**Figure 2:**
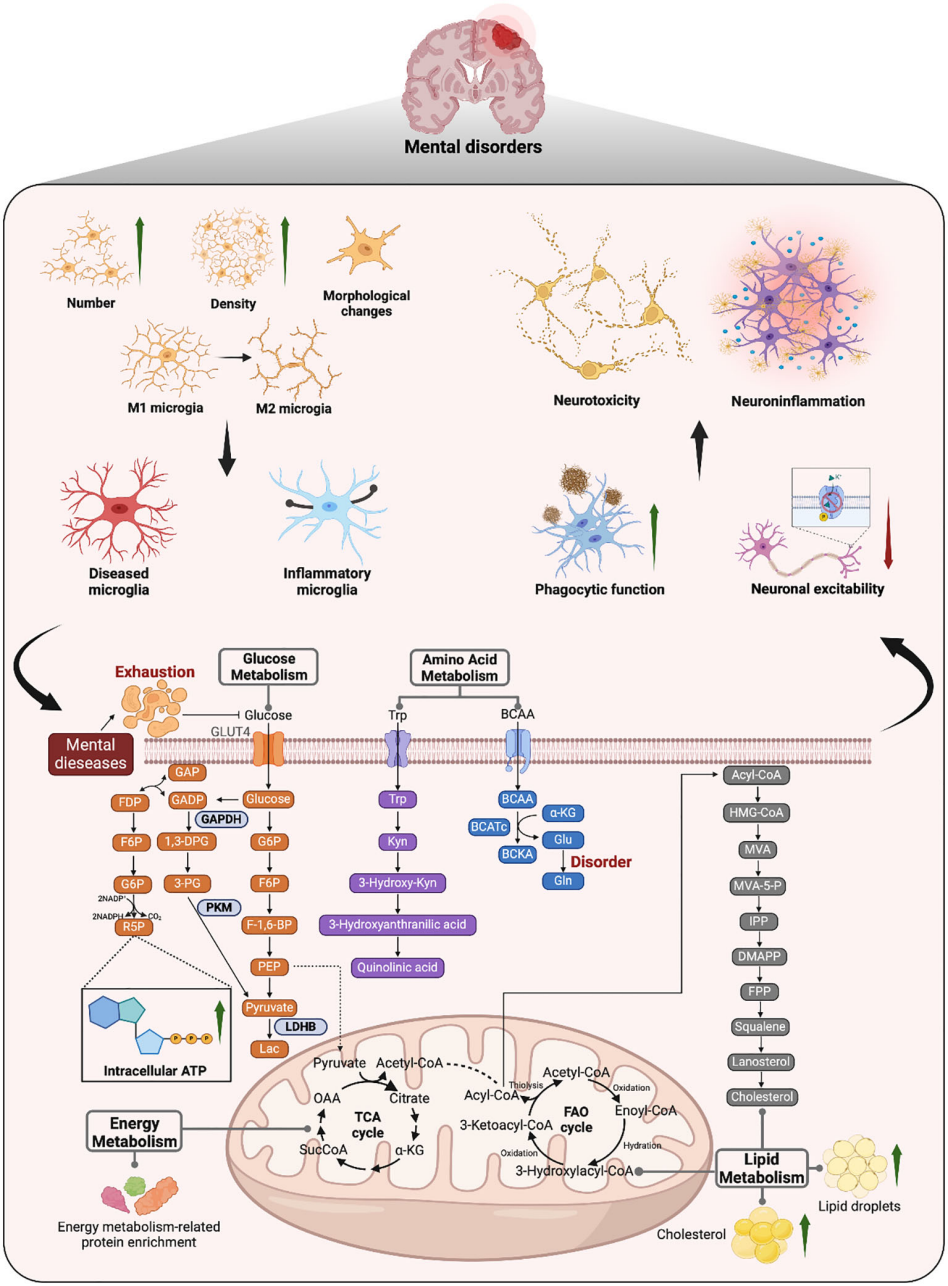
Proposed mechanisms by which microglial metabolic reprogramming contributes to the progression of mental disorders. Created with BioRender.com.

The manifestation of mental disorders triggers alterations in microglial activity, including changes in activation state, number, and morphology. These shifts in microglia inevitably result in changes at the cellular-level metabolic pathways, such as elevated glucose consumption or impaired uptake following microglial activation, which in turn affect microglial lipid metabolism by increased fatty acid synthesis and fatty acid oxidation; additionally, microglia undergo changes in amino acid metabolism pathways, which further influence microglial energy metabolism, including a decline in mitochondrial function. These metabolic pathway alterations within microglia often catalyze disease progression, intensifying conditions through mechanisms such as the activation of various enzymes involved in glycolysis, key enzymes in lipid metabolism, and the accumulation of cholesterol, all contributing to central nervous inflammation and neurotoxicity, thus promoting the advancement of related diseases. Research indicates that modulating these altered metabolites can often restore microglial function, thereby reducing conditions. For instance, knocking out the crucial glycolytic enzyme HK2 (89) or inhibiting fatty acid synthesis (172) can rejuvenate microglial phagocytic capability, ameliorating cognitive function impairment in AD, among other effects. The possible mechanism of how the metabolic pathway changes caused by microglia activity further promote the occurrence of mental diseases or symptoms is shown in **Figure 2**. Hence, investigating the metabolic pathway changes in microglia is crucial for understanding the mechanisms underlying mental disorders.

Step 1–Upstream triggers: Mental disorders (schizophrenia, AD/PD, depression) activate microglia through distinct triggers: inflammatory cytokines, protein aggregates (Aβ, tau, αSyn), or chronic stress (see Section 3.1.1.1).

Step 2–Metabolic reprogramming: Activated microglia shift their metabolism away from oxidative phosphorylation toward glycolysis and the pentose phosphate pathway (PPP). However, the direction of change differs by disease:

In schizophrenia, the kynurenine pathway is altered, increasing the kynurenic acid/quinolinic acid ratio.

In AD/PD, glycolysis is initially upregulated but later declines, accompanied by lipid droplet accumulation and cholesterol retention.

In depression, stress-induced CISD1 upregulation drives glycolytic activation.

Step 3–Functional consequences: These metabolic changes lead to excessive production of pro-inflammatory cytokines, neurotoxic molecules, and glutamate dysregulation, which in turn cause NMDA receptor dysfunction and neuronal apoptosis.

Step 4–Vicious cycle: Metabolic dysfunction further promotes microglial activation, creating a self-perpetuating loop that exacerbates neuroinflammation and disease progression.

#### 4.1.2 The metabolic pathway changes in astrocytes related to mental disorders

Similarly, alterations in astrocyte activity inevitably impact their metabolism. For example, in addition to microglia, astroglia activation also affects glutamate metabolism and affects psychiatric symptoms in patients with schizophrenia (113). In addition, research has shown that astrocytes expressing apolipoprotein E (APOE), a genetic risk factor for AD, increase glucose processing through the pentose phosphate pathway (PPP), facilitating gluconeogenesis and the biosynthesis of phospholipids and nucleotides compared to E3 astrocytes (195). The APOE4 variant is also associated with increased glycolytic activity in astrocytes (195). Further studies have identified that the APOE4 mutation in AD contributes to cholesterol accumulation in astrocytes, impairing mitochondrial homeostasis and oxidative phosphorylation (241–245). These findings link alterations in glucose, lipid, and energy metabolism pathways in astrocytes to the development of AD. Beyond AD, chronic stress-induced depression in mouse models (CUMS) reveals reduced TCA cycle flux in astrocytes, decreased metabolic activity (60), decreased rates of glucose oxidation, and alterations in the metabolic cycles of glutamate and GABA, as well as neurotransmitter cycles (249). Moreover, the expression of genes and proteins related to glutamate and GABA metabolism in astrocytes (such as glutamine synthetase, SLC1A2, and SLC1A3 glial glutamate transporters) is decreased in the brain cortex of major depressive disorder (MDD) patients (250), indicating that changes in glucose, amino acid, and energy metabolism pathways in astrocytes are associated with the onset of depression. These findings collectively affirm the close connection between mental disorders and changes in astrocytes metabolic pathways, the details in **Table 2**.

**Figure 3:**
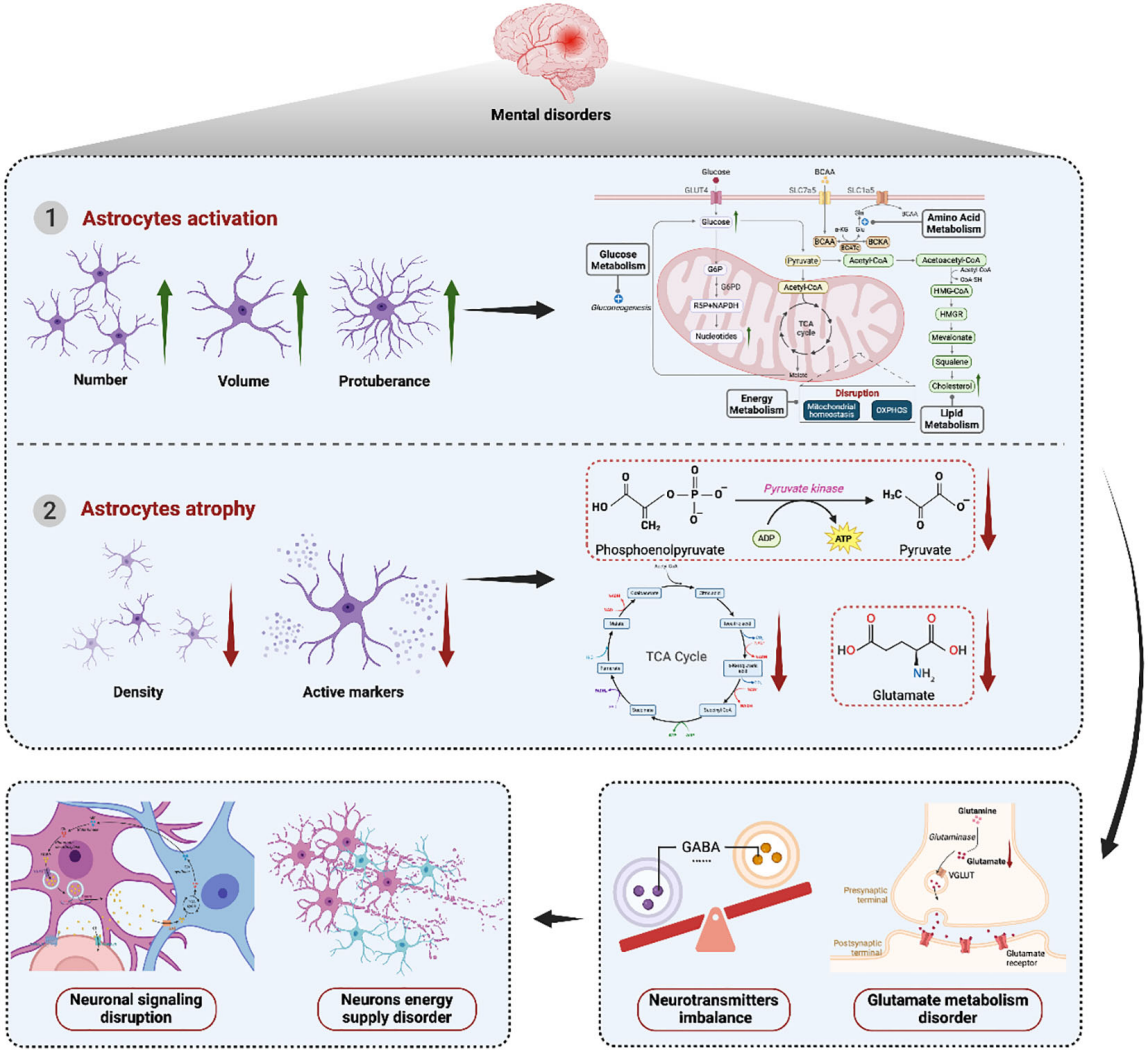
Proposed mechanisms by which astrocytic metabolic alterations contribute to the progression of mental disorders. Created with BioRender.com. This image contrasts two distinct astrocytic states observed across mental disorders: Reactive astrogliosis (increased cell volume, thickened processes) is prominent in AD and schizophrenia, often associated with APOE4-driven cholesterol accumulation, impaired mitochondrial homeostasis, and altered pentose phosphate pathway flux. Astrocytic atrophy (reduced density and marker expression) is more characteristic of depression and chronic stress, leading to decreased TCA cycle flux, reduced glutamate oxidation, and neurotransmitter cycle deficits. Disease-specific outcomes: In AD, astrocytic GABA accumulation impairs memory; in depression, reduced glutamine synthetase and glutamate transporters disrupt excitatory/inhibitory balance.

Previous studies have summarized the critical role of astrocytic metabolic pathway changes in the emergence of mental disorders: on one hand, these changes can lead to neurotransmitter imbalances, disrupting neuronal signal transmission and eliciting mental symptoms such as mood swings, anxiety, depression, hallucinations, and delusions (48). For instance, alterations in the putrescine degradation pathway in astrocytes can result in abnormalities in the neurotransmitter γ-aminobutyric acid (GABA), contributing to memory disorders in AD patients (196–198). On the other hand, astrocytic metabolic pathway changes can compromise neuronal energy supplies. Given astrocytes’ role in producing and releasing glutamate, a primary energy source for neurons, alterations in their metabolic pathways could decrease glutamate production and release, impacting neuronal energy availability (199) and impairing neuronal function (199, 200), thereby facilitating disease progression. The possible mechanism of how the metabolic pathway changes caused by astrocyte activity further promote the occurrence of mental diseases or symptoms is shown in **Figure 3**. Hence, investigating the metabolic pathway changes in astrocytes is crucial for understanding the mechanisms underlying mental disorders.

#### 4.1.3 Comparative perspective: how metabolic differences across diseases drive distinct immune outcomes

The three disorders examined in this review exhibit distinct metabolic signatures that translate into disease-specific patterns of immune activation. Schizophrenia is characterized by peripheral hyperglycemia, elevated triglycerides and monounsaturated/ω-6 polyunsaturated fatty acids, and increased kynurenic acid—an NMDA receptor antagonist. This metabolic environment favors a sustained, low-grade pro-inflammatory state in microglia, characterized by M1 polarization and impaired M2-mediated resolution of inflammation (96). The kynurenine pathway imbalance (increased KYNA) directly contributes to glutamatergic hypofunction and psychotic symptoms (113–119).

Alzheimer’s disease presents with reduced brain glucose uptake but increased glycolysis in early-stage microglia, which later transitions to metabolic exhaustion and lipid accumulation (56, 90). The presence of protein aggregates (Aβ, tau) continuously activates microglia, shifting them from a phagocytic disease-associated microglia (DAM) phenotype to a dysfunctional, senescent state that secretes pro-inflammatory cytokines but fails to clear pathology (101–104). This chronic activation is compounded by age-related metabolic changes, including lipid droplet accumulation in microglia and impaired mitochondrial oxidative phosphorylation (58, 178, 179).

Major depressive disorder is associated with regional hypometabolism in the prefrontal cortex, reduced serum cholesterol, and variable quinolinic acid levels depending on inflammatory state and brain region (73). In the hippocampus, microglial loss correlates with depressive behavior (92), while in the prefrontal cortex, activated microglia are increased (111). The underlying metabolic triggers—chronic stress, HPA axis dysregulation, and low-grade systemic inflammation—drive region-specific microglial responses that contribute to distinct symptom clusters (anhedonia, fatigue, cognitive deficits) (249, 250).

Thus, while metabolic reprogramming is a common theme across disorders, the direction, magnitude, and functional consequences differ fundamentally, reflecting the distinct etiologies and pathological processes of each disease.

### 4.2 The metabolic pathway changes in peripheral immune cells related to mental disorders

The complexity of mental disorders extends beyond the CNS, involving systemic metabolic changes. Alterations in the number, spatial distribution, and activation state of peripheral immune cells result in changes at the organelle and signaling molecule levels, ultimately affecting the body’s metabolite composition. These metabolites can circulate into the CNS and influence the emergence of related symptoms (201), highlighting the importance of understanding peripheral immune cell metabolic pathway changes in discussing mental disorder mechanisms.

#### 4.2.1 The metabolic pathway changes in T lymphocytes related to mental disorders

Changes in T lymphocyte subtypes can lead to changes in cell metabolism. For instance, pro-inflammatory T effector cells and T memory cells are inclined toward higher glycolysis rates, whereas anti-inflammatory T regulatory cells favor increased fatty acid oxidation (202, 203). Dysregulation in tryptophan metabolism has been observed in CD4^+^ T cell subtypes of schizophrenia patients (169, 170). Aging is associated with intrinsic metabolic defects in the mitochondria of CD4^+^ T cells, characterized by reduced glycolysis and a weakened induction of one-carbon metabolism. Activated T cells during aging show a decrease in metabolite levels across glycolysis, the pentose phosphate pathway, and the TCA cycle (171). Anxiety models induced by electrical foot shocks in mice reveal a severe reduction in glycolysis and oxidative phosphorylation (OXPHOS) in initial CD4^+^ T cells (180). This evidence indicates that the major metabolic pathways, including carbohydrate metabolism, lipid metabolism, amino acid metabolism energy metabolism, undergo significant changes in peripheral immune cells in the context of mental disorders, and the details in **Table 1**.

**Figure 4:**
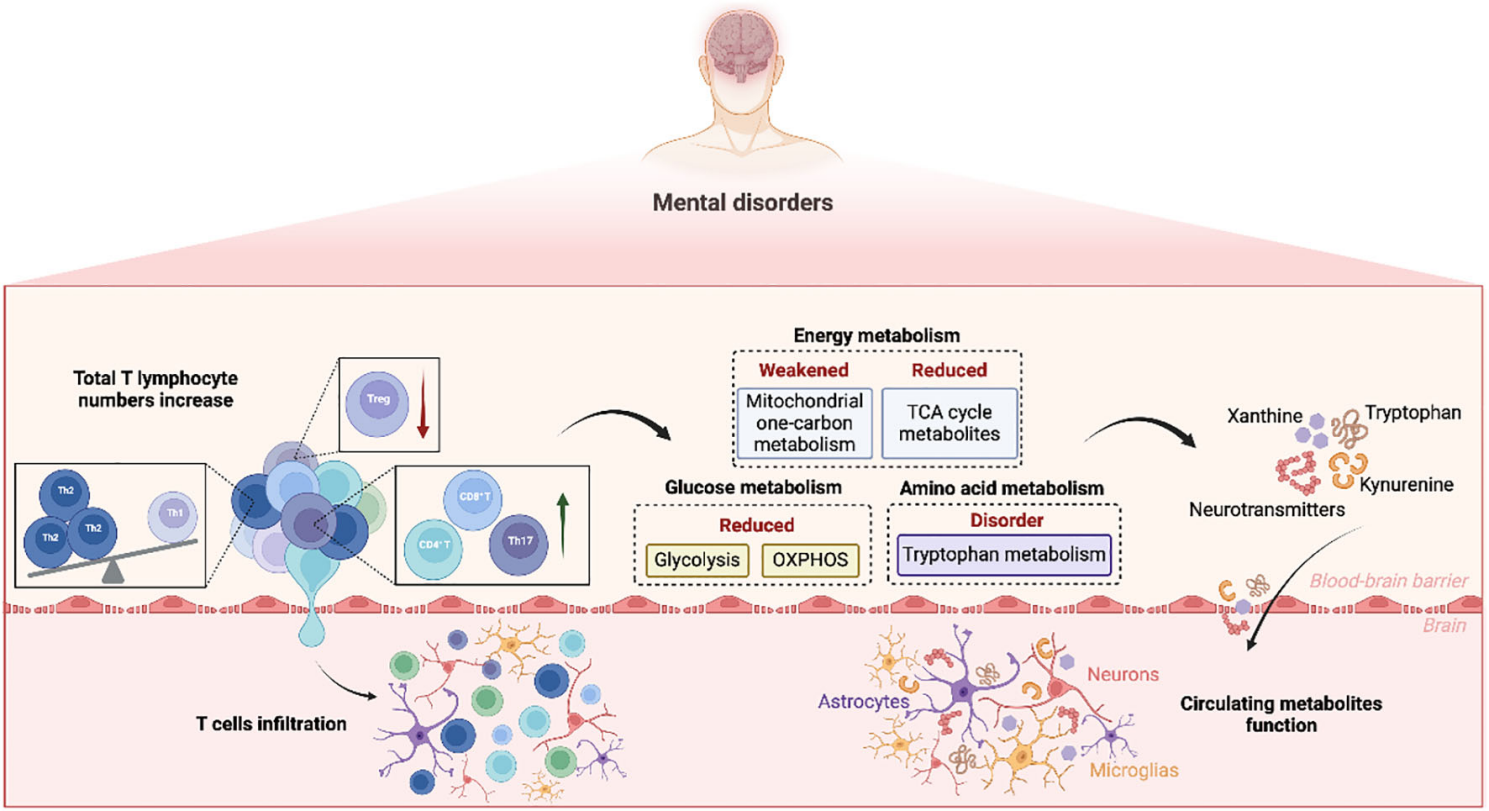
Proposed mechanisms by which T lymphocyte metabolic changes promote mental disorders. Created with BioRender.com. The image illustrates how peripheral T cell metabolic alterations (glycolysis, oxidative phosphorylation, one-carbon metabolism, tryptophan metabolism) generate circulating metabolites that cross the blood–brain barrier (BBB) and affect CNS glia and neurons. Disease-specific considerations: In schizophrenia, CD4^+^T cells exhibit tryptophan metabolism dysregulation with altered Th1/Th2 balance, and Treg cells are reduced or dysfunctional. In aging and AD, CD8^+^Teffector memory T cells (TEMRA) accumulate and infiltrate the CNS; aged T cells show intrinsic mitochondrial defects with reduced one-carbon metabolism. In depression and anxiety models, naive CD4^+^T cells display severe reduction in glycolysis and OXPHOS, and stress drives T cell migration to the brain. Common across disorders: Activated T cells release TNF-γ, inducing LAT transporters that carry tryptophan across the BBB, where it is metabolized into neuroactive kynurenine metabolites.

Under disease conditions, the integrity of the blood-brain barrier (BBB) is compromised, allowing circulating metabolites to enter the central nervous system. For instance, kynurenine can easily cross the BBB into the brain (67, 204), and neuroactive metabolites such as short-chain fatty acids (SCFAs) and neurotransmitters can also traverse a defective BBB (205). Activated CD4^+^ T cells releasing TNF-γ induce L-type amino acid transporters (LATs) to transport tryptophan (TRP) across the BBB (206). These infiltrating metabolites further influence central immune cells (such as microglia or astrocytes) and promote disease development. Tryptophan metabolites like kynurenine, once in the brain, are metabolized by perivascular macrophages, microglia, and astrocytes into neuroactive compounds, promoting the onset of depression (67). Mitochondrial fission in CD4^+^ T cells generating hypoxanthine affects oligodendrocytes in the left amygdala through the adenosine receptor A1, promoting anxiety behavior (180). An imbalance in CD4^+^ T cell subtypes Th1-Th2 can modify IDO (indoleamine 2,3-dioxygenase) and TDO (tryptophan 2,3-dioxygenase) activities, influencing tryptophan metabolism (169, 170), thus diverting tryptophan catabolism toward kynurenine (KYN), and its downstream metabolism toward either microglial quinolinic acid (Th1 response) or astrocytic kynurenic acid (Th2 response) (67, 169, 207), further exacerbating brain diseases. In summary, the progression of mental disorders is influenced by metabolites from peripheral T lymphocytes. The possible mechanism by which changes in metabolic pathways caused by T lymphocytes further promote the occurrence of mental illness or symptoms is shown in **Figure 4**.

#### 4.2.2 The metabolic pathway changes in other peripheral immune cells related to mental disorders

**Figure 5:**
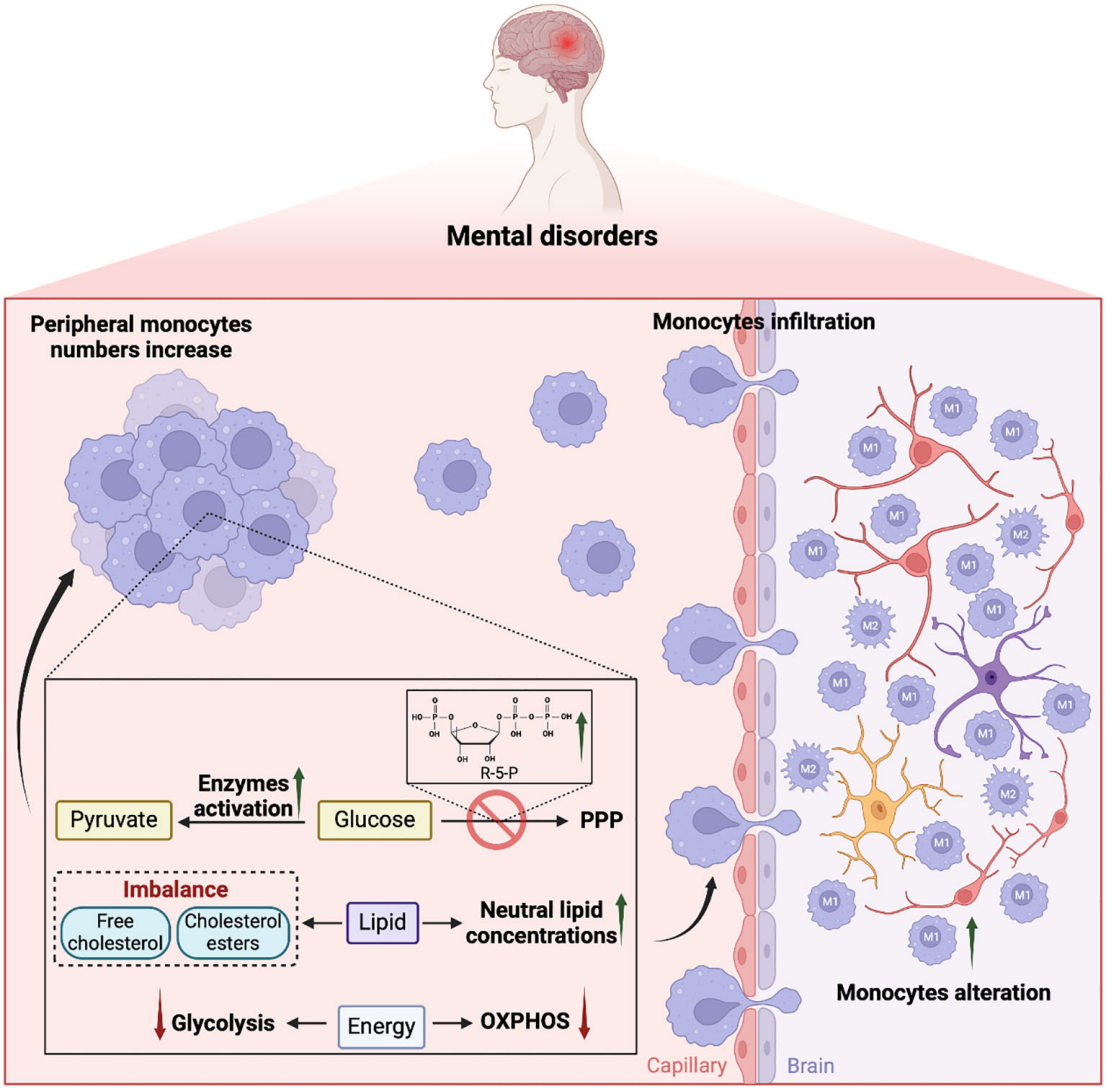
Proposed mechanisms by which peripheral monocyte/macrophage metabolic changes promote mental disorders. Created with BioRender.com. This image summarizes the metabolic alterations (glucose, lipid, energy) in peripheral monocytes/macrophages across mental disorders. Key differences by disease: In schizophrenia, peripheral blood mononuclear cells show increased glycolytic enzyme activity and pentose phosphate pathway dysregulation (elevated ribose 5-phosphate). In AD, monocytes display neutral lipid accumulation and cholesterol ester imbalance, with a shift from anti-inflammatory (M2) to pro-inflammatory (M1) phenotypes as disease progresses. In MDD, monocytes exhibit mitochondrial dysfunction and upregulation of cholesterol metabolism-related genes; chronic stress increases monocyte mobilization and BBB infiltration. A common consequence across disorders: Metabolically reprogrammed monocytes/macrophages can infiltrate the CNS (through a compromised BBB) and amplify neuroinflammation, contributing to disease progression.

Most studies on the metabolic changes of other peripheral immune cells related to mental diseases focus on the metabolic changes in individual mononuclear cells from peripheral blood. In schizophrenia patients, significant abnormalities are observed in the glucose metabolism of peripheral blood mononuclear cells, characterized by increased activity in several glucose metabolism enzymes, including pyruvate dehydrogenase complex, triosephosphate isomerase, glyceraldehyde-3-phosphate dehydrogenase, phosphoglycerate kinase 1, and phosphoglycerate mutase (25–29). Furthermore, the pentose phosphate pathway is disrupted in schizophrenia patients, particularly with a marked increase in 5-phosphoribosyl 1-pyrophosphate (15). In neurodegenerative diseases such as Alzheimer’s disease (AD), a notable rise in the concentration of neutral lipids occurs in the peripheral blood mononuclear cells of patients (52), alongside a documented imbalance between free cholesterol and cholesterol esters in cultured mononuclear cells from AD patients (53, 54), indicating a dysregulation in the lipid metabolism pathway. Additionally, circulating monocytes/macrophages in elderly patients exhibit a dependency on glucose, accompanied by a significant decrease in glycolysis and mitochondrial oxidative phosphorylation, which ultimately results in energy deficits (178, 179). Furthermore, mononuclear cells in patients with Major Depressive Disorder (MDD) demonstrate mitochondrial dysfunction, with several cholesterol metabolism genes markedly upregulated in these cells (181). Research has documented that peripheral monocytes/macrophages, along with their secreted cytokines, possess the capacity to traverse the blood-brain barrier and infiltrate into the central nervous system. This infiltration subsequently amplifies inflammatory responses and may contribute to the progression of psychiatric disorders (121). Consequently, alterations in the metabolic pathways of these monocytes/macrophages can significantly impact the quantity or functionality of the cells involved, as shown in **Figure 5**.

There is a paucity of reports on metabolic changes in peripheral B lymphocytes, NK cells, and neutrophils in mental disorders. Research has indicated increased activity of indoleamine 2,3-dioxygenase (IDO) in NK cells during conditions such as cerebral ischemia, Parkinson’s disease (PD), Huntington’s disease (HD), and other neurodegenerative diseases (175). Additionally, studies show that aging impacts the metabolism of peripheral NK and B cells. For instance, there is a significant reduction in the production of inositol triphosphate (IP3) in NK cells of older individuals (174), and aging is associated with reduced glycolysis and defects in mitochondrial energy production in B cells (173). However, further research is needed to determine whether these metabolic alterations are linked to the development of mental disorders.

To sum up, the onset of mental disorders leads to changes in the activity of peripheral immune cells, including fluctuations in the numbers of T lymphocytes, B lymphocytes, NK lymphocytes, monocytes/macrophages, and neutrophils. These variations in immune cell activity result in alterations in cellular-level metabolic pathways, encompassing carbohydrate, lipid, amino acid, and energy metabolism, and the details in **Table 1**. The metabolites generated by these metabolic changes enter the circulation and pass through the compromised BBB into the CNS, where they affect the activity of central glial cells (microglia, astrocytes, oligodendrocytes) or directly impact neuronal function, thereby facilitating the progression of mental disorders. Dummy **Table 3**.

**Table 3:** Comparative summary of immunometabolic reprogramming across mental disorders.

Feature	Schizophrenia (SCZ)	Alzheimer’s disease (AD)/PD	Major depressive disorder (MDD)
Microglial activation trigger	Peripheral/central inflammation (cytokines)	Aβ/tau/αSyn aggregates (DAMPs)	Chronic stress, glucocorticoids
Microglial metabolic shift	Increased glycolysis, PPP activation	Glycolysis↑ early, then dysfunction; lipid droplet accumulation	CISD1-mediated glycolysis↑
Astrocytic state	Mixed (increased number in some regions; decreased GFAP in others)	Reactive astrogliosis (A1 phenotype)	Atrophy (reduced density and markers)
Peripheral T cell changes	CD4^+^↑, Treg↓ or dysfunctional, Th17↑	CD8^+^ TEMRA↑, infiltration into CNS	CD4^+^↑, Treg suppressed by stress
Monocyte/macrophage changes	Glycolytic enzyme↑, PPP↑, infiltration into brain	M1/M2 imbalance, neutral lipid accumulation	Mitochondrial dysfunction, cholesterol gene↑
Key amino acid pathway	Kynurenine pathway (↑KYNA)	Kynurenine pathway (↑QUIN)	Kynurenine pathway (variable QUIN/KYNA)
Lipid signature	↑TG, ↑MUFA/PUFA, ↑ribose-5-P	Cholesterol accumulation, lipid droplets	↓Cholesterol
Energy metabolism	TCA cycle activation early; mitochondrial dysfunction	OXPHOS↓, mitochondrial dysfunction	TCA cycle↓ in astrocytes

### 4.3 Distinct effects of metabolic reprogramming patterns on immune inflammation

Metabolic reprogramming is not a monolithic process; different patterns of metabolic change within immune cells produce distinct inflammatory outcomes. Here, we categorize the major patterns observed across mental disorders and their effects on immune inflammation.

#### 4.3.1 Glycolytic shift (Warburg-like effect)

Activation of microglia and peripheral monocytes is accompanied by a metabolic shift from oxidative phosphorylation (OXPHOS) to aerobic glycolysis—a phenomenon analogous to the Warburg effect in cancer cells (66, 176, 177, 183–189). This glycolytic bias has several pro-inflammatory consequences: (i) increased production of lactate, which serves as a signaling molecule that further promotes inflammatory gene expression; (ii) enhanced flux through the pentose phosphate pathway, supplying nucleotides and NADPH for cytokine synthesis and reactive oxygen species (ROS) production; and (iii) reduced mitochondrial respiration, leading to decreased ATP production efficiency but allowing rapid metabolic adaptation to immune challenges. In M1-polarized microglia, this glycolytic shift is particularly pronounced and directly correlates with the production of IL-1β, TNF-α, and IL-6 (190). In schizophrenia and early-stage AD, this pattern dominates and is associated with acute neuroinflammatory responses.

However, chronic reliance on glycolysis comes at a cost. Prolonged glycolytic activation leads to accumulation of glycolytic intermediates, such as methylglyoxal, which promote protein glycation and cellular stress. Moreover, sustained glycolytic demand depletes cellular NAD^+^, impairing sirtuin-mediated anti-inflammatory pathways and epigenetic regulation of immune genes (180).

#### 4.3.2 Lipid metabolic reprogramming

Alterations in lipid metabolism produce distinct inflammatory phenotypes depending on the direction of change. Increased fatty acid oxidation (FAO) —observed in M2-polarized microglia—supports anti-inflammatory functions, tissue repair, and resolution of inflammation. M2 microglia increase mitochondrial FAO and fatty acid synthesis, utilizing lipids as an alternative energy source when glucose availability is limited (190). In contrast, lipid droplet accumulation—characteristic of aged microglia and late-stage AD—is associated with a dysfunctional, pro-inflammatory state. Lipid droplet-accumulating microglia (LDAM) upregulate genes related to lipogenesis and FAO while downregulating key lipid degradation enzymes, resulting in increased ROS production and sustained secretion of pro-inflammatory cytokines (58).

Cholesterol accumulation—driven by APOE4 in AD microglia—represents a distinct lipid-related inflammatory mechanism. Accumulated cholesterol affects specific potassium ion channels on neuronal membranes, inhibiting neuronal firing and reducing excitability while also promoting microglial inflammation (91). Similarly, in MDD, reduced serum cholesterol is consistently observed, but the functional consequences for immune cells remain less clear. One hypothesis is that membrane cholesterol depletion alters lipid raft composition and disrupts Toll-like receptor signaling, potentially leading to impaired immune responses (208).

#### 4.3.3 Mitochondrial dysfunction and energy failure

Mitochondria are central hubs integrating metabolic and immune signaling. Impaired oxidative phosphorylation (OXPHOS) —observed across all three disorders—leads to reduced ATP production, limiting energy-dependent immune functions such as phagocytosis and antigen presentation. In AD microglia, chronic exposure to Aβ causes mitochondrial toxicity, resulting in metabolic dysfunction and reduced clearance of protein aggregates (105). In schizophrenia, mitochondrial dysfunction has been implicated as a core feature, with decreased TCA cycle flux and impaired energy production contributing to neuronal vulnerability (46).

More importantly, mitochondrial dysfunction triggers the release of mitochondrial DNA (mtDNA) and other damage-associated molecular patterns (DAMPs), which activate the NLRP3 inflammasome and drive IL-1β/IL-18 production—a process that amplifies neuroinflammation independently of the initial trigger (209). In MDD, circulating monocytes show evidence of mitochondrial dysfunction with upregulation of cholesterol metabolism-related genes, and this impairment correlates with treatment resistance (181).

#### 4.3.4 Amino acid pathway reprogramming

The kynurenine pathway of tryptophan metabolism exemplifies how a single metabolic pathway can produce divergent inflammatory outcomes depending on the direction of reprogramming. Increased quinolinic acid (QUIN) —an NMDA receptor agonist—promotes excitotoxicity, oxidative stress, and neuroinflammation. This pattern is observed in AD, PD, and a subset of MDD patients with high inflammatory burden (39–45, 73). Increased kynurenic acid (KYNA) —an NMDA receptor antagonist—produces a different outcome: while neuroprotective in some contexts, excessive KYNA impairs glutamatergic transmission and contributes to cognitive deficits and psychotic symptoms, as observed in schizophrenia (113–119).

Glutamate metabolism also exhibits disease-specific patterns. While elevated glutamate is observed in schizophrenia (thalamus, basal ganglia, medial temporal lobe) and MDD (serum), the underlying mechanisms differ. In schizophrenia, microglial activation leads to impaired glutamate-to-glutamine conversion and increased KYNA production, favoring NMDA receptor hypofunction (113). In MDD, the relationship is more complex: some studies report elevated serum glutamate (70, 71), while chronic unpredictable mild stress (CUMS) mouse models show reduced glutamate and glutamine (60), suggesting that disease stage, stress chronicity, and brain region critically determine the direction of change.

#### 4.3.5 Summary of pattern–outcome relationships

**Table 4** provides a systematic comparison of how different metabolic reprogramming patterns affect immune inflammation across disorders.

**Table 4:** Comparative effects of metabolic reprogramming patterns on immune inflammation across mental disorders.

Metabolic pattern	Immune outcome	Key disorder association
Glycolytic shift (↑glycolysis, ↑PPP)	Pro-inflammatory (M1) polarization; ↑IL-1β, TNF-α, IL-6; ↑ROS; ↑kynurenine flux	Schizophrenia, early AD, stress-induced depression
Lipid droplet accumulation (LDAM)	Dysfunctional, pro-inflammatory; ↓phagocytosis; sustained cytokine secretion	Aging, late-stage AD
↑Fatty acid oxidation (FAO)	Anti-inflammatory (M2) polarization; tissue repair; resolution of inflammation	Homeostatic microglia, recovery phase
Cholesterol accumulation	Impaired neuronal firing; microglial inflammation; ↓excitability	AD (APOE4 carriers)
Mitochondrial OXPHOS impairment	↓ATP; ↓phagocytosis; mtDNA release → NLRP3 activation → ↑IL-1β/IL-18	Schizophrenia, MDD (treatment-resistant), aging
↑Quinolinic acid (QUIN)	Excitotoxicity; oxidative stress; NMDA agonism → neuroinflammation	AD, PD, MDD (high inflammation subset)
↑Kynurenic acid (KYNA)	NMDA antagonism; cognitive deficits; psychosis	Schizophrenia
Reduced OXPHOS + ↓glycolysis in T cells	Impaired immune regulation; ↓Treg function; hypoxanthine release	Anxiety, stress-induced depression

The ↑ symbol indicates Upregular or Enhance, and the ↓ symbol indicates downregular or inhibit.

## 5 Therapeutic strategies targeting immunometabolic reprogramming

The mechanistic evidence presented in preceding sections—demonstrating that metabolic reprogramming of both central and peripheral immune cells drives neuroinflammation and disease pathology—raises the compelling question of whether these metabolic pathways can be therapeutically targeted. While the field remains in its early stages, a growing body of preclinical and emerging clinical evidence suggests that immunometabolic interventions hold promise for mental disorders. This section reviews pharmacological approaches targeting key metabolic pathways, lifestyle and dietary interventions, and the major challenges that must be overcome to translate these strategies into clinical practice.

### 5.1 Pharmacological targeting of metabolic pathways

#### 5.1.1 Glycolytic pathway

Given the central role of glycolysis in microglial activation across mental disorders, glycolytic enzymes have emerged as attractive therapeutic targets. In the AD brain, microglial hexokinase 2 (HK2)—the enzyme that catalyzes the first committed step of glycolysis and supports inflammatory responses—is significantly upregulated (17). Importantly, HK2 also displays non-metabolic activities that extend its inflammatory role beyond glycolysis (17). Codocedo et al. demonstrated that HK2 antagonism affects microglial phenotypes and disease progression in a gene-dose-dependent manner: complete loss of HK2 fails to improve pathology by exacerbating inflammation, while haploinsufficiency reduces pathology in 5xFAD mice. This finding carries critical therapeutic implications—partial antagonism of HK2 (e.g., through the inhibitor lonidamine) may be effective in slowing disease progression by modulating NF-κB signaling via IKBα, whereas complete inhibition would be counterproductive (17). Similarly, compounds that inhibit pyruvate kinase M2 (PKM2) or activate AMP-activated protein kinase (AMPK) have shown significant potential in neurodegenerative diseases (210). For instance, the natural PKM2 inhibitor shikonin attenuates rotenone-driven neuroinflammation through dual mechanisms—suppressing glycolysis and reducing neurotoxic metabolite formation—correlating with significant inhibition of the microglial pro-inflammatory cascade (211). SGLT2 inhibitors such as enavogliflozin enhance microglial mitochondrial function and Aβ phagocytosis via AMPK activation, establishing them as promising therapeutic candidates for AD (212). GLP-1 receptor agonists also alleviate AD-related phenotypes through AMPK activation in microglia, inhibiting neuroinflammation and promoting Aβ phagocytosis (213).

In stress-induced depression models, the mitochondrial protein CISD1 is upregulated in microglia of the medial prefrontal cortex after chronic stress, promoting NADH oxidation to NAD^+^ and accelerating glycolysis (87). Pharmacological inhibition and genetic knockdown of CISD1 significantly ameliorate depressive-like behavior in mice (87). Notably, pioglitazone—a PPARγ agonist already approved for type 2 diabetes—exerts antidepressant effects by inhibiting NADH oxidation through a CISD1-dependent pathway in microglia. PPARγ agonism also induces the neuroprotective phenotype of microglia and ameliorates depression-like behaviors in chronic mild stress-treated mice (87). These findings establish a robust experimental foundation for repurposing pioglitazone as an antidepressant, though well-powered clinical trials are needed.

#### 5.1.2 Kynurenine pathway

The kynurenine pathway of tryptophan metabolism represents another major therapeutic frontier. Given that pathway dysregulation—characterized by imbalanced quinolinic acid (neurotoxic) and kynurenic acid (neuroprotective) ratios—is implicated across schizophrenia, AD/PD, and MDD, targeting key enzymatic nodes offers disease-modifying potential.

For depression, while early attention focused on IDO1 inhibition, recent evidence suggests that IDO induction is not a major event in MDD; instead, its metabolic consequences may be masked and overridden by upregulation of kynurenine monooxygenase (KMO), the gateway to production of neuroactive metabolites (214). KMO appears to be activated in MDD by certain proinflammatory cytokines, and antidepressants with anti-inflammatory properties may block this activation. Importantly, the antidepressant ketamine can dock (bind) to KMO, suggesting that KMO inhibition may underlie some of its rapid effects. Preclinical studies have confirmed that both IDO1 inhibitors and TDO inhibitors attenuate depressive-like behaviors in inflammation- and stress-induced mouse models (214). Novel indole-based IDO1 inhibitors have been designed and show antidepressant potential in preclinical evaluation (215). Ginsenoside compound K has been identified as a brain-penetrant KMO inhibitor that alleviates corticosterone-induced depressive-like behaviors and restores the neuroactive kynurenine pathway balance by modulating the quinolinic acid/kynurenic acid ratio (216). The enrichment of KMO in thalamic neurons—as opposed to cortical microglia—offers new insights into the neuroanatomical basis of KMO function and the mechanism of antidepressant interventions.

For schizophrenia, where increased kynurenic acid (an NMDA receptor antagonist) contributes to cognitive deficits and psychotic symptoms, strategies that modulate the kynurenine pathway toward a more balanced state remain in early preclinical development.

#### 5.1.3 Immunometabolic checkpoints

The concept of “immunometabolic checkpoints”—molecules that integrate immune and metabolic signaling—has gained traction. CD38, an NAD^+^-consuming enzyme, has emerged as a key immunometabolic checkpoint in AD. In individuals with familial AD, a unique immunometabolic signature in circulating CD4^+^ T cells features elevated CD38 expression preceding symptom onset (217). Treatment with an anti-CD38 antibody in 5xFAD mice restored T cell glucose metabolism and mitochondrial mass, improved metabolic fitness and cognitive performance, and attenuated local neuroinflammation by dampening IL-1β-dependent TH17 pathogenic immunity. Comprehensive profiling revealed that targeting CD38 abrogates meningeal TH17 immunity and cortical IL-1β, breaking the negative feedback loop between these two compartments (217). These findings suggest CD38 as both a pre-symptomatic biomarker for early AD diagnosis and a therapeutic target that could be used alone or in combination with other immunotherapies.

### 5.2 Lifestyle and dietary interventions

#### 5.2.1 Ketogenic diet

The ketogenic diet (KD)—a low-carbohydrate, moderate-protein, high-fat regimen that induces ketosis—has garnered renewed interest as a metabolic therapy for mental disorders (18). In pharmacological mouse models, a KD regimen resulted in complete restoration of normal behaviors independent of strict caloric restriction. An open-label KD study in the 1950s reported improvement in schizophrenia symptoms, and at least seven additional case reports have found robust improvements or complete resolution of schizophrenia symptoms. A retrospective study recently found robust and significant improvements in schizophrenia symptoms in 10 schizoaffective disorder patients treated with a KD (218). A case report documented a 48-year-old woman with treatment-resistant schizophrenia and metabolic syndrome who completed a 5-week medical KD in an inpatient setting, with the diet reversing insulin resistance and greatly improving psychiatric symptoms (219). By inducing ketosis and altering brain metabolism, KD may improve psychiatric symptoms by targeting underlying metabolic and neurochemical dysfunctions.

However, several limitations must be acknowledged (1): to date, no published double-blind randomized controlled trials have evaluated KD effects in schizophrenia (2); adherence to KD is challenging, particularly in populations with executive dysfunction (3); long-term safety and metabolic consequences remain uncertain; and (4) which patients are most likely to benefit remains unknown, highlighting the need for biomarker-guided patient selection.

#### 5.2.2 Exercise and lifestyle modification

Exercise may be particularly beneficial for patients with immunometabolic depression (IMD)—a subtype characterized by inflammatory and metabolic dysregulations that may respond less well to standard antidepressants. The MOTAR study suggested that exercise more effectively targets the IMD dimension than antidepressants. Persons with immuno-metabolic depression (approximately 20–30% of people with depression) are at higher risk for cardiometabolic diseases and seem to respond less well to standard antidepressant treatment. Interventions targeting inflammation, metabolism, or lifestyle may be more effective treatment options for these individuals, in line with principles of precision psychiatry (220).

### 5.3 Challenges and limitations

Despite the promise of immunometabolic targeting, substantial challenges impede clinical translation. First, patient stratification remains inadequate. Outcomes from clinical trials evaluating immunometabolic targeted interventions have been inconsistent, largely due to limited stratification of participants based on underlying biology. Immunologic and metabolic approaches are prominent in contemporary psychiatric discourse, yet translation into clinical practice remains variable. The immuno-metabolic depression subtype affects only 20–30% of depressed patients; treating unselected populations with immunometabolic agents would dilute effect sizes and may lead to erroneous conclusions about efficacy (221). Second, the directionality and context-dependence of metabolic modulation pose fundamental challenges. As demonstrated by the HK2 example, complete inhibition of a metabolic target can exacerbate pathology while partial inhibition provides benefit (17). Similarly, glycolysis is protective in early-stage AD microglia (supporting Aβ phagocytosis) but becomes maladaptive with chronic activation. Therapeutic windows may be narrow and disease-stage dependent, complicating clinical trial design. Third, target selection between central and peripheral compartments remains unresolved. Should immunometabolic therapies target the CNS directly (requiring brain-penetrant compounds), the peripheral immune system (with downstream effects on the CNS), or both? The complexity of immune–brain communication pathways—involving the BBB, meningeal spaces, and peripheral-to-central signaling—means that optimal targeting strategies remain unclear. Fourth, safety concerns are non-trivial. Metabolic targets such as HK2, PKM2, and AMPK are ubiquitous “housekeeping” enzymes; systemic inhibition carries risks of off-target effects on non-immune tissues. While pioglitazone and metformin have established safety profiles, newer agents targeting immunometabolic checkpoints require rigorous safety evaluation. Fifth, the interplay between antipsychotic/antidepressant medications and immunometabolism is poorly understood. Many psychotropic drugs have metabolic side effects that may confound or counteract immunometabolic interventions. Conversely, some antidepressants may exert part of their therapeutic effects through immunometabolic modulation—for example, ketamine’s binding to KMO—raising questions about how to design add-on trials that appropriately control for background medication effects (222, 251).

### 5.4 Future directions

Addressing these challenges will require a multi-pronged approach. Biomarker development is paramount: identifying peripheral blood or CSF markers that predict immunometabolic dysfunction and treatment response will enable patient stratification for clinical trials. The Psychosis Immune Mechanism Stratification (PIMS) project, which combines clinical, cognitive, and immunological profiling to define biologically meaningful subgroups of patients stratified by plasma IL-6 who may benefit from immune-targeted treatments, exemplifies this precision psychiatry approach (252).

Multi-omics integration—combining genomics, transcriptomics, proteomics, and metabolomics—will accelerate target discovery and validation. Combination strategies that target multiple nodes of the immunometabolic network (e.g., glycolysis inhibition plus kynurenine pathway modulation) may prove more effective than single-agent approaches.

Finally, repurposing of existing drugs with established safety profiles—such as metformin (which regulates the TCA cycle in schizophrenia), pioglitazone, GLP-1 receptor agonists, and statins—offers a more rapid path to clinical translation than *de novo* drug development. The ongoing trial of semaglutide in individuals with schizophrenia spectrum disorders exemplifies this strategy.

In summary, while the therapeutic targeting of immunometabolic reprogramming in mental disorders remains in its infancy, the mechanistic rationale is compelling. Success will depend on rigorous patient stratification, careful attention to the context-dependence of metabolic modulation, and the integration of immunometabolic approaches into the broader framework of precision psychiatry.

## 6 Conclusions and future directions

This review systematically integrates current evidence demonstrating that immunometabolic reprogramming is not merely a passive consequence but an active driver of pathology across schizophrenia, Alzheimer’s disease, Parkinson’s disease, and major depressive disorder. We have delineated three core principles that emerge from the collective evidence.

First, metabolic reprogramming follows disease-specific trajectories. While a glycolytic shift and mitochondrial dysfunction are common across disorders, the upstream triggers—inflammatory cytokines in schizophrenia, protein aggregates in AD/PD, and chronic stress in depression—dictate distinct microglial and astrocytic metabolic phenotypes. These range from reactive M1 polarization in early schizophrenia to lipid-droplet-accumulating dysfunctional microglia in aging and late-stage AD, and region-specific metabolic depression in stress-related disorders. This heterogeneity cautions against a one-size-fits-all therapeutic approach and underscores the necessity of disease-stage and subtype stratification.

Second, peripheral-central immune crosstalk operates through a metabolically active interface. We propose a working model wherein metabolic alterations in peripheral immune cells (T cells, monocytes/macrophages) generate circulating metabolites that, upon crossing a compromised blood-brain barrier, modulate the functional states of CNS glia. This peripheral-to-central metabolic relay represents a critical, and previously underexplored, axis linking systemic metabolic disturbances—such as hyperglycemia, dyslipidemia, and tryptophan pathway dysregulation—to central neuroinflammation and cognitive/behavioral symptoms. The kynurenine pathway exemplifies this axis, with peripheral Th1/Th2 imbalances dictating whether central tryptophan metabolites shift toward neurotoxic quinolinic acid or neuroprotective kynurenic acid.

Third, metabolic and immune dysfunctions form a self-perpetuating vicious cycle. Metabolically reprogrammed microglia produce pro-inflammatory cytokines that further impair neuronal metabolism and synaptic plasticity, while mitochondrial dysfunction in both central and peripheral immune cells releases damage-associated molecular patterns that perpetuate NLRP3 inflammasome activation. Breaking this cycle requires interventions that target not merely one node but the interconnected metabolic-immune network.

Looking forward, we identify several pressing directions. The integration of multi-omics approaches—single-cell transcriptomics, metabolomics, and proteomics—with longitudinal clinical phenotyping holds promise for identifying robust biomarkers that can stratify patients by immunometabolic subtype. Such stratification will be essential for the success of mechanism-based clinical trials, as the immunometabolic depression subtype affects only a subset (approximately 20–30%) of depressed patients. Furthermore, the development of brain-penetrant or peripherally-restricted metabolic modulators that achieve the desired immunomodulatory effect without disrupting essential housekeeping functions remains a major pharmacological challenge.

Finally, we emphasize that immunometabolic reprogramming offers a unifying framework that transcends traditional diagnostic boundaries. By linking peripheral metabolic derangements to central immune dysfunction, this perspective not only deepens our mechanistic understanding of mental disorders but also opens avenues for novel biomarker development and targeted interventions. The path forward lies in translating these mechanistic insights into clinical practice through rigorous patient stratification and thoughtfully designed trials that account for the context-dependence and stage-specificity of metabolic modulation.
